# Fatty acid-binding protein 4 is a therapeutic target for septic acute kidney injury by regulating inflammatory response and cell apoptosis

**DOI:** 10.1038/s41419-022-04794-w

**Published:** 2022-04-11

**Authors:** Bo Wang, Jun Xu, Qian Ren, Lu Cheng, Fan Guo, Yan Liang, Letian Yang, Zhouke Tan, Ping Fu, Liang Ma

**Affiliations:** 1 Kidney Research Institute, Division of Nephrology, West China Hospital of Sichuan University, 610041 Chengdu, China; 2 Research Core Facility of West China Hospital, Sichuan University, 610041 Chengdu, China; 3 Division of Nephrology, ZunYi Medical University Affiliated Hospital, 563003 ZunYi, China

**Keywords:** Acute kidney injury, Chronic kidney disease

## Abstract

Sepsis is a systemic inflammatory state in response to infection, and concomitant acute kidney injury (AKI) significantly increases morbidity and mortality. Growing evidence suggests that fatty acid-binding protein 4 (FABP4) is critically involved in kidney diseases, while its role in septic AKI remains unknown. Here, FABP4 was mainly upregulated in renal tubular epithelial cells (RTECs) following cecal ligation and puncture (CLP)- or lipopolysaccharide (LPS)-induced septic AKI. FABP4 inhibition by genetic deletion or BMS309403 treatment both attenuated kidney dysfunction and pathological injury in CLP- or LPS-treated mice. Notably, RTEC-specific deletion of FABP4 also showed similar renoprotective effects. Moreover, FABP4 inhibition alleviated inflammation and apoptosis in CLP-injured kidneys and LPS-stimulated mouse tubular epithelial cells. Mechanistically, TLR4 blockage improved sepsis-induced kidney injury, as well as suppressed c-Jun phosphorylation and FABP4 expression, where c-Jun knockdown also inhibited LPS-stimulated FABP4 level. Meanwhile, FABP4 inhibition reduced the elevated phosphorylated c-Jun, while the levels of TLR4 and MyD88 were uninfluenced. Collectively, the increased FABP4 in RTECs is dependent on TLR4/c-Jun signaling activation and contributes to kidney injury, by forming a positive feedback loop with c-Jun to aggravate inflammation and apoptosis in septic AKI. Thus, FABP4 may be a therapeutic target for septic AKI.

## Abstract (web summary)

Upregulation of tubular FABP4 in septic AKI is dependent on TLR4/c-Jun signaling activation, and FABP4 mediates sepsis-induced RTEC injury, likely by forming a positive feedback loop with c-Jun to aggravate inflammation and apoptosis.

## Graphical abstract

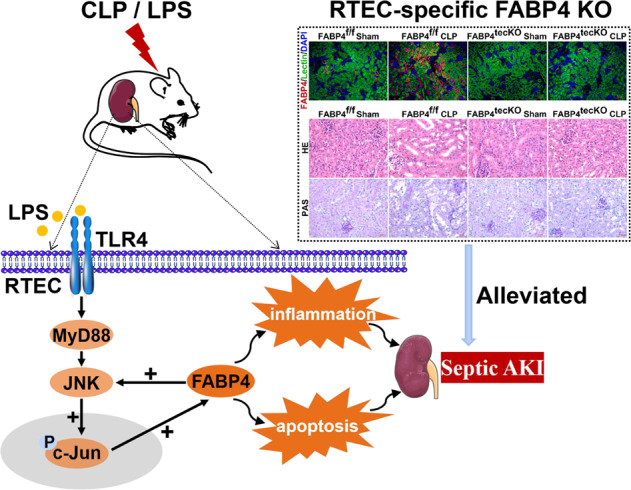

## Introduction

Sepsis is a complex syndrome characterized by organ dysfunction caused by a dysregulated host response to infection, and the kidney is the most commonly affected organ [1, 2]. Up to 50% of acute kidney injury (AKI) is attributable to sepsis, and up to 60% of sepsis patients occur AKI [2–4]. In addition to high morbidity, septic AKI patients have a worse prognosis and a higher mortality than sepsis patients without AKI [1, 3]. Although efforts have been devoted to study septic AKI pathogenesis, the mechanisms remain largely unknown, which results in no effective and specific treatment [1–3].

Although the mechanisms of septic AKI are complex and multifactorial, tubular injury has received extensive attention where inflammation and cell apoptosis are two prominent features [1–5]. During sepsis, renal tubular epithelial cells (RTECs) could be directly damaged by inflammatory responses [1, 4]. Injured RTECs could in turn modulate and amplify intrarenal inflammation and further cause tubular apoptosis, leading to RTECs loss [2, 4, 5]. Up to now, cecal ligation and puncture (CLP) and lipopolysaccharide (LPS) are two classical sepsis models [6–8]. CLP is considered the gold standard of sepsis [9], LPS is a component of the outer membrane of Gram-negative bacteria [10], while Gram-negative bacteria sepsis accounts for up to 50% of sepsis in-hospital mortality worldwide [11]. Toll-like receptor 4 (TLR4), which is activated by LPS, together with its downstream mediator MyD88, has significant roles in inflammation and cell apoptosis of septic AKI [12–14]. In particular, as the downstream effector of TLR4/MyD88 signaling, c-Jun NH2-terminal kinase (JNK)/c-Jun cascade also emerges as a central regulator in kidney diseases [15–18].

Fatty acid-binding protein 4 (FABP4) is well known for its pathogenic effect in metabolic diseases such as atherosclerosis and diabetes mellitus [19–21]. Recently, FABP4 has also emerged as a critical player in inflammation and apoptosis in various pathological processes [22–24]. We previously found that FABP4 expression increased in injured RTECs of ischemia/reperfusion-, rhabdomyolysis-, and cisplatin-induced AKI, and FABP4 inhibition by BMS309403 alleviated tubular injury, while the mechanisms of FABP4 upregulation were poorly understood [25–27]. Yet, whether FABP4 is implicated in septic AKI needs to be revealed.

In the study, we aimed to explore the role of FABP4 in septic AKI. We found that sepsis-induced FABP4 expression in RTECs was dependent on TLR4/c-Jun signaling activation, and FABP4 mediated tubular damage in septic AKI, by forming a positive feedback loop with c-Jun to amplify inflammation and apoptosis. Our results suggested that FABP4 may be a promising therapeutic target for septic AKI.

## Methods

### Chemicals and antibodies

BMS309403 (S6622) and TAK242 (S7455) were purchased from Selleck (Shanghai, China). LPS (L8880, purity≥99%) from *Escherichia coli* 055:B5 was obtained from Solarbio (Beijing, China). Primary antibodies were exhibited in Table S1. HRP-conjugated secondary antibodies were purchased from Thermo Fisher Scientific (Waltham, MA, USA).

### Animals

The animal procedures and experimental protocols were approved by the Experimental Animal Ethics Committee of West China Hospital of Sichuan University. Male C57BL/6J mice, FABP4 knockout (KO) mice described previously [25], RTEC-specific FABP4 KO mice (Fig. S1), and TLR4 KO mice were subjected to CLP or LPS injection to induce septic AKI. All the animals were divided randomly into groups with 6 mice in each group. The sample size was determined according to previous experiments. The details of the generation of RTEC-specific FABP4 KO mice and animal studies are provided in the Supplementary Methods.

### Renal function analysis

Blood samples were coagulated and subsequently centrifuged at 3000 rpm/min for 20 min at room temperature to gather serum. Serum creatinine (Scr) and blood urea nitrogen (BUN) levels were measured using a biochemical automatic analyzer (Mindray BS-240, Shenzhen, China). The AKI model was successfully established when the Scr level of the CLP group rose up to 2 times of their sham littermates.

### Histologic examination

Kidney tissues were fixed in 10% neutral buffered formalin, embedded in paraffin, and sectioned at 4 μm thickness. Kidney sections were stained with hematoxylin and eosin (HE) or periodic acid-Schiff (PAS) after deparaffinization and rehydration. The sections were viewed by an AxioCamHRc digital camera (Carl Zeiss, Jena, Germany) at ×200 and ×400 magnifications. HE staining was assessed at ×200 magnification with tubular injury scores in a blinded manner. Briefly, tubular injury was scored by two independent pathology doctors without knowing grouping. Tubular injury score was evaluated on a scale of 0–4, with 0, 1, 2, 3, and 4 corresponding to 0%, <25%, 26–50%, 51–75%, ≥76% of injured/damaged renal tubules, respectively [25].

### Transmission electron microscopy

Kidney tissues were fixed in cold 2.5% glutaraldehyde for 2 h at 4 °C and then treated with standard procedures, including dehydration, osmosis, embedding, sectioning, and staining, and finally visualized on a Hitachi microscope (H-7650, Calgary, AB, Japan) at ×8000, ×12,000, and ×20,000 magnifications.

### Immunohistochemistry

Formalin-fixed, dehydrated, paraffin-embedded kidney sections (4 μm) were deparaffinized, rehydrated, and antigen-retrieved. The slides were then blocked by 2.5% normal goat serum and incubated with primary antibodies anti-FABP4 (1:200, 12802-1-AP, Proteintech Group, Chicago, USA), anti-TLR4 (1:200, Ab13556, Abcam, MA, USA), and anti-phospho-c-Jun (1:200, ET1608-4, HuaAn Biotechnology, Hangzhou, China) at 4°C. The slides were washed thrice in PBS and stained using VECTASTAIN ABC Kit (Vector, Burlingame, CA, USA). Images were captured using an AxioCamHRc digital camera (Carl Zeiss, Jena, Germany) at ×200 and ×400 magnifications with ZEN 2012 microscopy software (blue edition). The positive area of immunohistochemistry staining was calculated at ×200 magnification with ImageJ software (version 1.51, Wayne Rasband, National Institutes of Health, USA).

### Immunofluorescence staining

OCT-embedded kidney sections (4 μm) were incubated with phosphate-buffered saline (PBS) containing 5% horse serum for 1 h at room temperature to block non-specific binding sites. Then the specimens were incubated with primary antibody anti-FABP4 (1:200, 12802-1-AP, Proteintech Group, Chicago, USA) in a humidified chamber overnight at 4°C. After washing, the secondary antibody (1:500, 111-025-003, Jackson ImmunoResearch, West Grove, PA, USA) was used for 1 h. Fluorescein-labeled *Lotus tetragonolobus lectin* (LTL) (1:400, FL-1321, Vector Laboratories, CA, USA) was applied for identifying proximal tubules. The samples were washed again and then stained with DAPI (1:500, D8200, Solarbio, Beijing, China) and finally sealed with coverslips. Images were acquired by an AxioCamHRc digital camera (Carl Zeiss, Jena, Germany) at ×200 magnification with ZEN 2012 microscopy software (blue edition).

### TUNEL staining

Formalin-fixed, dehydrated, paraffin-embedded kidney sections (4 μm) were mounted on glass slides. Terminal deoxynucleotidyl transferase-mediated dUTP nick-end labeling (TUNEL) assay (G3250, Promega, Madison, Wisconsin, USA) in kidney sections was performed as previously described [25]. The slides were visualized with an AxioCamHRc digital camera (Carl Zeiss, Jena, Germany) at ×200 magnification and the TUNEL-positive cells were counted from 10 randomly picked images for each sample.

### RNA-sequencing analysis

Three kidney samples from each group (Sham and CLP group) were randomly selected for sequencing. Total RNA was extracted with Trizol reagent (Invitrogen, Carlsbad, CA, USA), followed by sample integrity, quality, and purity examination. Library construction and sequencing were performed by LC-BIO Bio-tech Ltd (Hangzhou, China). The libraries were then sequenced with the Illumina NovaSeq^TM^ 6000 platform and paired-end reads were generated with read length 2 × 150 bp.

### Western blot analysis

Western blot analysis was performed as described earlier [27]. Densitometry analysis was carried out using ImageJ software (version 1.51, Wayne Rasband, National Institutes of Health, USA) and GAPDH was used as the internal reference protein.

### Quantitative real-time PCR analysis

Three samples per group were randomly selected for quantitative real-time PCR (RT-qPCR). Total RNA isolation and RT-qPCR were performed as described previously [27]. The primers for detected genes are listed in Table S2. The relative gene quantities were calculated by the 2^−∆∆Ct^ method and GAPDH was used as the internal reference gene.

### Cell culture and treatments

Mouse RTECs (TCMK-1, ATCC^®^ CCL-139^TM^, Beijing bnbio Co. Ltd, China) were cultured in MEM/EBSS medium (SH30024.01, Hyclone, Beijing, China) containing 10% fetal bovine serum (SH30084.03, Hyclone, Beijing, China) and 1% penicillin-streptomycin solution (SV30010, HyClone, Beijing, China) at 37 °C under humidified atmosphere of 5% CO_2_ and 95% air. The cells were recently authenticated and tested negative for mycoplasma contamination. The Cells were serum-starved in medium containing 0.5% serum for 24 h and then treated with 100 μg/ml LPS for another 24 h. BMS309403 was used to TCMK-1 cells at 10 μM for 30 min prior to LPS treatment. The details of the transfection of FABP4 siRNA, TLR4 siRNA, c-Jun siRNA, and negative control (NC) siRNA in TCMK-1 cells are provided in the Supplementary Methods.

### Cell viability assay

Cell viability was determined by the Cell Counting Kit-8 assay (CCK-8, APExBIO, Houston, TX, USA) according to the manufacturer’s instructions. Briefly, TCMK-1 cells in logarithmic growth phase were seeded in 96-well culture plates at a density of 5000 cells/well. After treating with BMS309403 or LPS, a 10 μl CCK-8 solution was added to each well and incubated in dark for 1 h at 37°C. In the end, the absorbance at 450 nm was detected using a microplate reader (Synergy Mx, Biotek, Vermont, USA).

### Annexin V-FITC/propidium iodide assay

Apoptosis in cultured cells was assessed by the Annexin V-FITC apoptosis analysis kit (AO2001-02P-H, SUNGENE, Tianjin, China). In short, TCMK-1 cells were harvested at indicated time points, resuspended with a binding buffer, and finally stained with 5 μl Annexin V-FITC and 5 μl propidium iodide (PI) in dark for 15 min at room temperature. At last, apoptotic cells were analyzed by a flow cytometer (Beckman Cytoflex, Beckman Coulter Australia Pty Ltd., Lane Cove, NSW, Australia).

### Statistical analysis

The random number table method was used for random allocation. All experiments were repeated at least three times. Data were represented as mean ± SD. The number of biological replicates was presented by individual data points in each bar graph. Statistical differences between two groups were performed by two-tailed Student’s *t* test (for parametric data) or Mann–Whitney *U* test (for non-parametric data), and comparisons between multiple groups were analyzed with one-way ANOVA (for one experimental parameter) or two-way ANOVA (for two experimental parameters) followed by Tukey’s multiple comparisons test using GraphPad Prism 6.01 (GraphPad Software, San Diego, CA, USA). *P* value less than 0.05 was considered significant.

## Results

### FABP4 deficiency and BMS309403 treatment both attenuated septic AKI

**Fig. 1:**
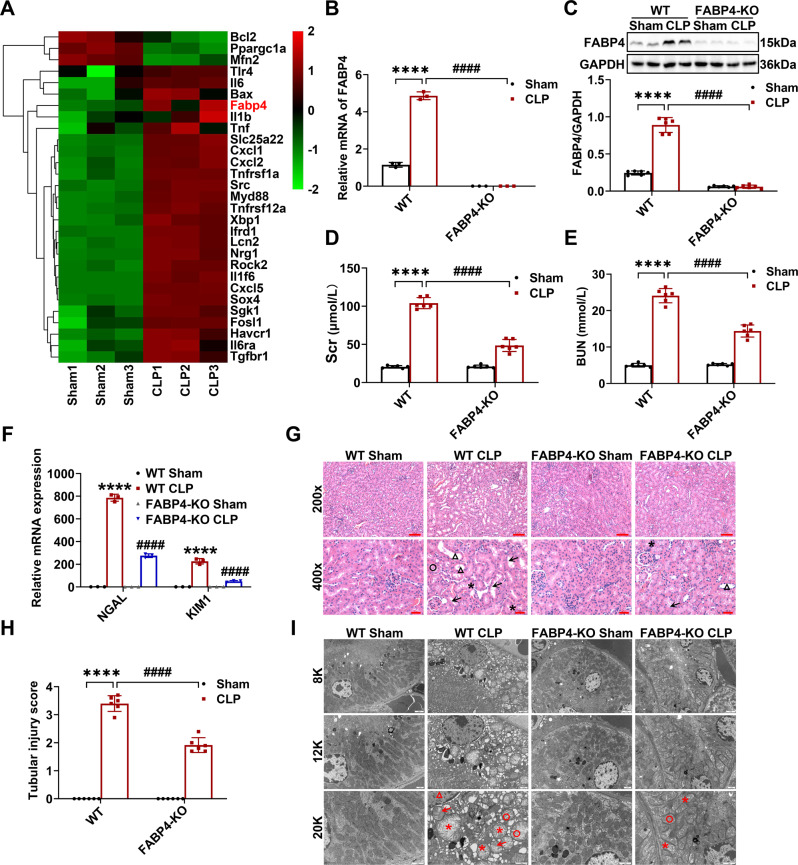
FABP4 deficiency ameliorated CLP-induced septic AKI in mice. C57BL/6J, FABP4 KO, and WT mice (male, 8–10 weeks old) were subjected to CLP or sham surgery and killed 16 h later. **A** Representative heatmap of differentially expressed genes in the kidneys of C57BL/6J mice between sham and CLP group (*n* = 3). **B** Quantitative real-time PCR analysis of FABP4 in kidney tissues (*n* = 3). **C** Western blotting of FABP4 and quantification by densitometry normalized with GAPDH in the kidneys (*n* = 6). **D** Serum creatinine (Scr) and (**E**) BUN in different groups of mice (*n* = 6). **F** Quantitative real-time PCR analysis of NGAL and KIM1 in kidney tissues (*n* = 3). **G** Representative HE staining micrographs (×200, scale bar = 50 μm; ×400, scale bar = 20 μm) and (**H**) tubular injury scores of kidney tissues (*n* = 6). Triangle: tubular dilatation; Asterisk: tubular swelling; Circle: cast formation; Arrow: loss of brush border. **I** Representative subcellular structures of renal tubular epithelial cells (RTECs) from different groups of mice collected by transmission electron microscope (TEM) (×8000, scale bar = 2 μm; ×12,000, scale bar = 1 μm; ×20,000, scale bar = 1 μm). Triangle: chromosome condensation; Asterisk: mitochondrial swelling; Circle: mitochondrial cristate fused; Arrow: mitochondrial cristate disappeared. All data are represented as mean ± SD; ^****^P < 0.0001, versus WT Sham; ^####^*P* < 0.0001, versus WT CLP.

Firstly, we found that FABP4 expression was markedly upregulated in kidneys of CLP-induced septic AKI mice, as indicated by RNA-Seq analysis (Fig. 1A), RT-qPCR (Fig. 1B), and western blotting (Fig. 1C). Notably, RNA-Seq exhibited that the genes involved in TLR4 signaling, inflammation, and apoptosis were upregulated in kidneys of CLP group compared to those of sham group, while Bcl2 was downregulated (Fig. 1A).

Next, to elucidate the role of FABP4 in septic AKI, FABP4 KO mice were subjected to CLP. FABP4 KO mice were phenotypically normal and had no appreciable defects in renal function or morphology (Fig. 1D–I). However, FABP4 deficiency led to a marked decrease in CLP-induced FABP4 expression (Fig. 1B, C) and ameliorated septic AKI, as evidenced by a reduction of Scr and BUN (Fig. 1D, E), and decreased kidney neutrophil gelatinase-associated lipocalin (NGAL) and kidney injury molecule 1 (KIM1) mRNA levels (Fig. 1F). Meanwhile, in comparison with WT mice, FABP4 KO mice displayed an effective improvement in pathological damage characterized by tubular dilatation, swelling, cast formation, and loss of brush border after CLP (Fig. 1G, H). In addition, the ultra-structural changes in RTECs of WT mice after CLP were observed, including chromosome condensation, mitochondrial swelling, mitochondrial cristate fused and even disappeared, which were alleviated by FABP4 KO (Fig. 1I).

**Fig. 2:**
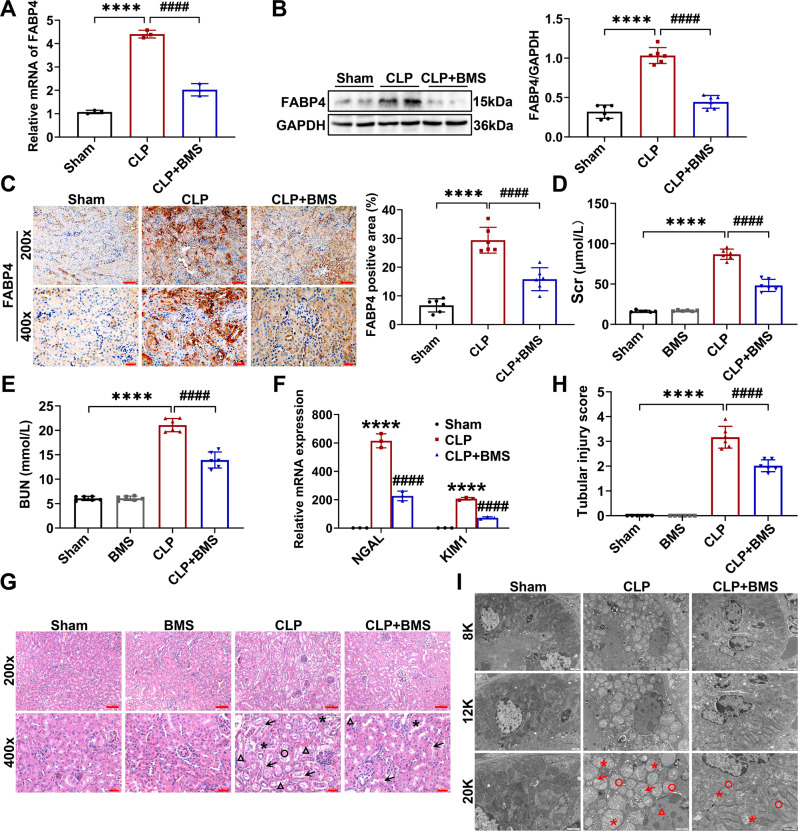
FABP4 inhibitor BMS309403 treatment alleviated CLP-induced septic AKI in mice. C57BL/6J and BMS309403 (BMS)-treated C57BL/6J mice (male, 8–10 weeks old) were subjected to CLP or sham surgery and killed 16 h later. **A** The mRNA expression of FABP4 in the kidneys analyzed by quantitative real-time PCR analysis (*n* = 3). **B** Protein expression of kidney FABP4 detected by western blotting and quantified by densitometry (*n* = 6). **C** Representative micrographs and quantitative analysis of immunochemistry staining of FABP4 in kidney tissue sections (×200, scale bar = 50 μm; ×400, scale bar = 20 μm). **D** Serum creatinine (Scr) and (**E**) BUN concentrations in different groups of mice (*n* = 6). **F** The mRNA expression of NGAL and KIM1 analyzed by quantitative real-time PCR analysis in kidney tissues (*n* = 3). **G** Representative images of HE staining (×200, scale bar = 50 μm; ×400, scale bar = 20 μm) and **H** tubular injury scores of kidney tissues (*n* = 6). Triangle: tubular dilatation; Asterisk: tubular swelling; Circle: cast formation; Arrow: loss of brush border. **I** Representative electron micrographs of renal tubular epithelial cells (RTECs) from different groups of mice (×8000, scale bar = 2 μm; ×12,000, scale bar = 1 μm; ×20,000, scale bar = 1 μm). Triangle: chromosome condensation; Asterisk: mitochondrial swelling; Circle: mitochondrial cristate fused; Arrow: mitochondrial cristate disappeared. Data are shown as mean ± SD; ^****^P < 0.0001, versus Sham; ^####^*P* < 0.0001, versus CLP.

To investigate whether FABP4 inhibitor provides a renoprotective effect on septic AKI, the mice were orally administered BMS309403 at a dose of 40 mg/kg/d for 3 days before CLP or LPS injection. As shown in Fig. 2A–C, BMS309403 administration significantly reduced the rise of kidney FABP4 mRNA and protein levels induced by CLP. As growing evidence suggested that RTEC played a crucial role in AKI [1, 2, 4], we further verified that FABP4 was mainly upregulated in renal tubules following CLP (Fig. 2C). BMS309403 treatment markedly decreased the CLP-induced elevation of Scr and BUN with relatively good safety (Fig. 2D, E), lowered the NGAL and KIM1 mRNA levels in injured kidneys (Fig. 2F), and mitigated renal morphologic (Fig. 2G, H) and ultra-structural (Fig. 2I) damage. Similarly, FABP4 expression was also induced by LPS injection, and then inhibited by BMS309403 treatment in LPS-injured kidneys (Fig. S2A, B). Meanwhile, the LPS-induced kidney dysfunction (Fig. S2C, D) and tubular injury (Fig. S2E–G) were also alleviated by BMS309403 treatment. Taken together, FABP4 was induced in RTECs by sepsis, while both genetic and pharmacologic inhibition of FABP4 attenuated septic AKI.

### Inhibition of FABP4 reduced kidney inflammation and apoptosis in CLP-induced septic AKI

**Fig. 3:**
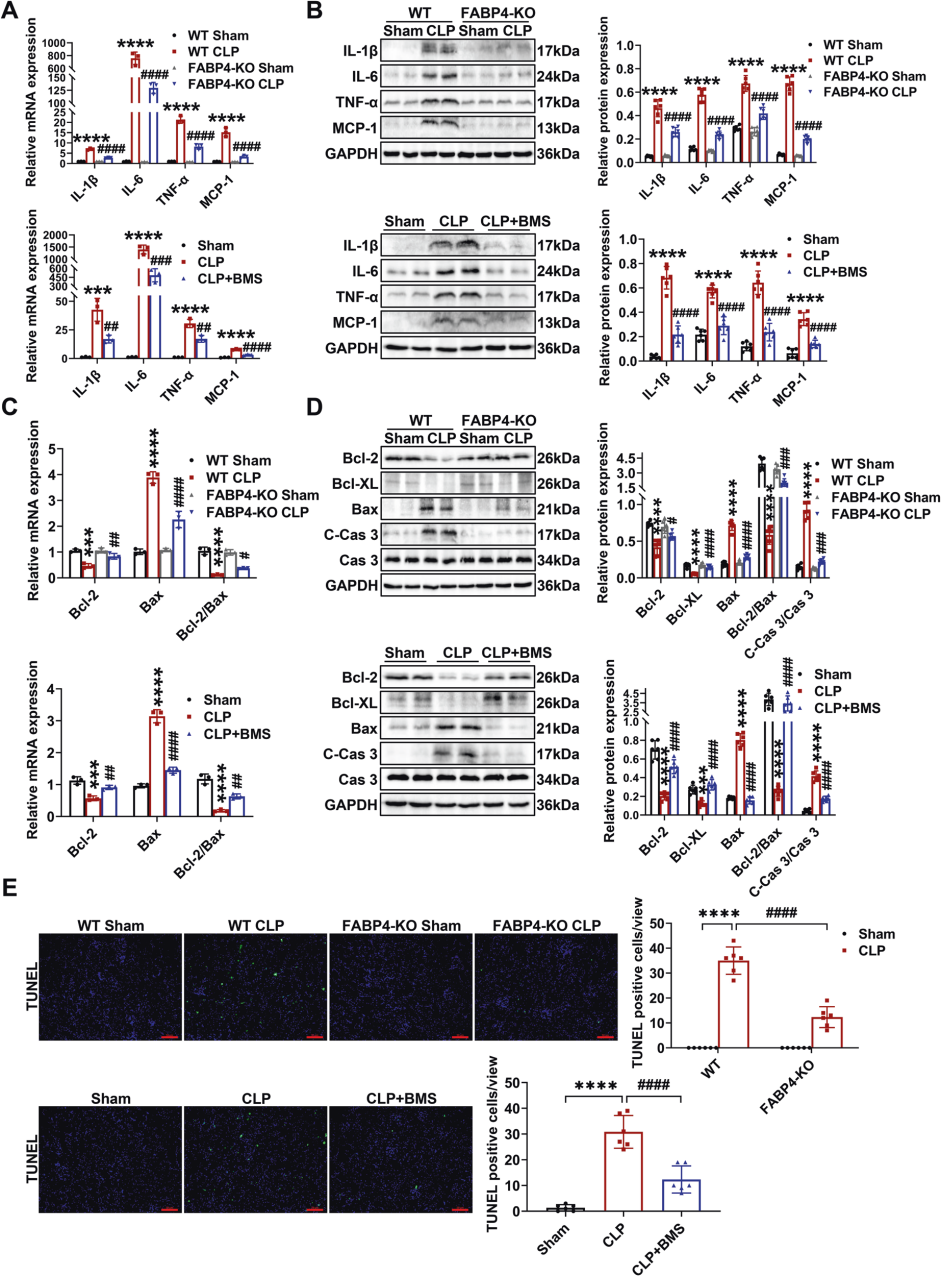
Genetic or pharmacologic inhibition of FABP4 alleviated CLP-induced kidney inflammation and apoptosis in mice. C57BL/6J, FABP4 inhibitor BMS309403 (BMS)-treated C57BL/6J, FABP4 KO, and WT mice (male, 8–10 weeks old) were subjected to CLP or sham surgery and killed 16 h later. **A** The mRNA levels of inflammatory cytokines including IL-1β, IL-6, TNF-α, and MCP-1 in kidney tissues measured by quantitative real-time PCR (*n* = 3). **B** The protein levels of inflammatory cytokines including IL-1β, IL-6, TNF-α, and MCP-1 in the kidneys examined by western blotting and quantified by densitometry (*n* = 6). **C** The mRNA levels of apoptotic markers including Bcl-2 and Bax in renal tissues measured by quantitative real-time PCR (*n* = 3). **D** The protein levels of apoptotic markers including Bcl-2, Bcl-XL, Bax, Caspase 3 (Cas 3), and cleaved-caspase 3 (C-Cas 3) in the kidneys analyzed by western blotting and quantified by densitometry (*n* = 6). **E** Representative images of TUNEL staining (×200, scale bar = 50 μm) and quantification of TUNEL-positive cells in kidney cortex (*n* = 6). All data are represented as mean ± SD; ^***^P < 0.001, ^****^P < 0.0001 for WT CLP versus WT Sham, or for CLP versus Sham; ^#^*P* < 0.05, ^##^*P* < 0.01, ^###^*P* < 0.001, ^####^*P* ^<^ 0.0001 for FABP4-KO CLP versus WT CLP, or for CLP + BMS versus CLP.

Kidney inflammation and apoptosis are two notable characteristics in the pathogenesis of septic AKI [2, 5, 28]. We found that the levels of IL-1β, IL-6, TNF-α, and MCP-1 in kidneys were dramatically induced by CLP (Fig. 3A, B). Meanwhile, pro-apoptotic cleaved-caspase 3 (C-Cas 3) and Bax were upregulated in kidneys of CLP mice, in association with a marked increase in the number of TUNEL-positive cells, while the levels of anti-apoptotic Bcl-2 and Bcl-XL were markedly decreased (Fig. 3C–E). In general, the abovementioned abnormalities of proinflammatory factors, apoptotic markers, and TUNEL-positive cell number were significantly attenuated by FABP4 deficiency or BMS309403 treatment (Fig. 3A–E), which indicated that FABP4 inhibition could suppress kidney inflammation and apoptosis induced by CLP.

### CLP-induced upregulation of FABP4 was dependent on TLR4 signaling activation

**Fig. 4:**
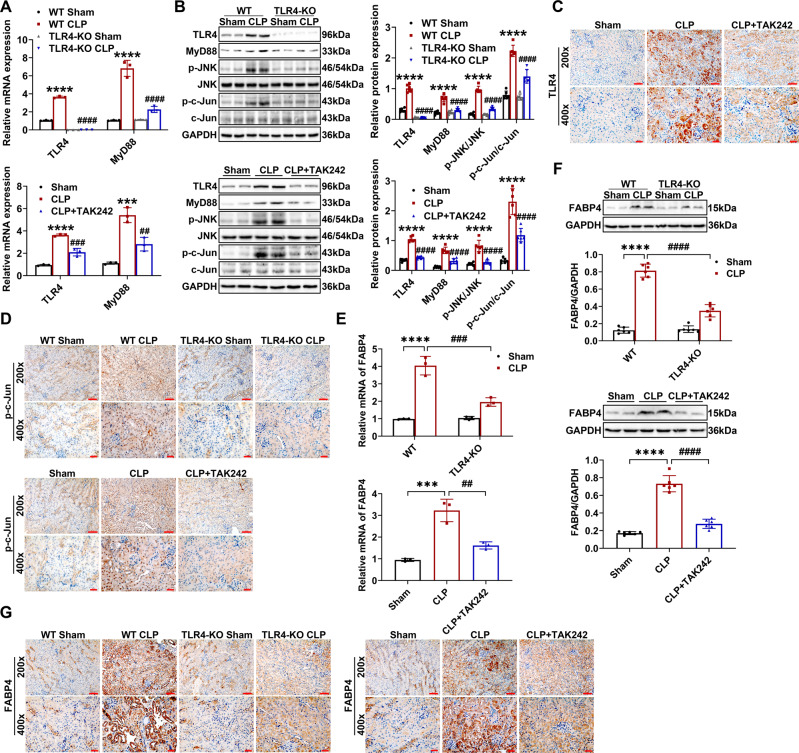
CLP-induced upregulation of FABP4 was mediated by TLR4 signaling in injured kidneys. C57BL/6J, TLR4 inhibitor TAK242-treated C57BL/6J, TLR4 KO, and WT mice (male, 8-10 weeks old) were subjected to CLP or sham surgery and killed 16 h later. **A** Quantitative real-time PCR analysis of TLR4 and MyD88 in kidney tissues (*n* = 3). **B** Western blot analysis of TLR4, MyD88, p-JNK, JNK, p-c-Jun, and c-Jun in the kidneys (*n* = 6). Immunochemistry staining of (**C**) TLR4 and (**D**) p-c-Jun in kidney tissues (×200, scale bar = 50 μm; ×400, scale bar = 20 μm). **E** Quantitative real-time PCR analysis of FABP4 in renal tissues (*n* = 3). **F** Western blot analysis of FABP4 in the kidneys (*n* = 6). **G** Immunochemistry staining of FABP4 in kidney tissue sections (×200, scale bar = 50 μm; ×400, scale bar = 20 μm). Data are shown as mean ± SD; ^***^P < 0.001, ^****^P < 0.0001 for WT CLP versus WT Sham, or for CLP versus Sham; ^##^*P* < 0.01, ^###^*P* < 0.001, ^####^*P* < 0.0001 for TLR4-KO CLP versus WT CLP, or for CLP + TAK242 versus CLP.

TLR4/MyD88/JNK signaling regulates c-Jun, a vital intracellular modulator of inflammation and cell death in kidney under different pathological conditions [12, 15, 18]. Besides, c-Jun was once reported as a transcription factor targeting FABP4 [23]. Combining the kidney RNA-Seq results in which TLR4 and MyD88 were upregulated by CLP (Fig. 1A), we thus determined whether TLR4/MyD88/JNK/c-Jun signaling contributes to FABP4 induction in CLP-injured kidneys. As confirmed by RT-qPCR and western blotting, the elevated expression of TLR4, MyD88, phosphorylated JNK (p-JNK), and phosphorylated c-Jun (p-c-Jun) in CLP-injured kidneys was inhibited by TLR4 KO or TLR4 inhibitor TAK242 administration (Fig. 4A, B). In addition, we found that TLR4 and nuclear p-c-Jun were mostly induced in renal tubules following CLP, whereas reduced by TLR4 KO or TAK242 treatment (Fig. 4C, D).

Further, TLR4 KO and TAK242 treatment both showed significant renoprotective (Fig. S3A–F), anti-inflammatory (Fig. S4A, B), and anti-apoptotic (Fig. S4C–E) effects. At the same time, the results of RT-qPCR, western blotting, and immunohistochemistry revealed that the elevation of renal FABP4 due to CLP could be remarkably suppressed through TLR4 KO or TAK242 treatment (Fig. 4E–G). In general, these data indicated that CLP-induced FABP4 upregulation in RTECs was dependent on TLR4 signaling activation.

### Inhibition of FABP4 improved inflammation and apoptosis in LPS-stimulated TCMK-1 cells

To further confirm whether FABP4 inhibition by siRNA or BMS309403 could repress inflammation and apoptosis in vitro, we used LPS to stimulate mouse renal tubular epithelial (TCMK-1) cells. As delineated in Fig. S5A–H, the LPS-induced FABP4 expression, TLR4/MyD88/JNK/c-Jun signaling activation, inflammation (marked by IL-1β, IL-6, and TNF-α), and apoptosis (marked by cleaved-caspase 3) in a dose- and time-dependent manner (0-200 μg/ml and 0–48 h), and peaked at 100 μg/ml and 24 h, respectively. Hence, we finally chose 100 μg/ml LPS to stimulate TCMK-1 cells for 24 h.

**Fig. 5:**
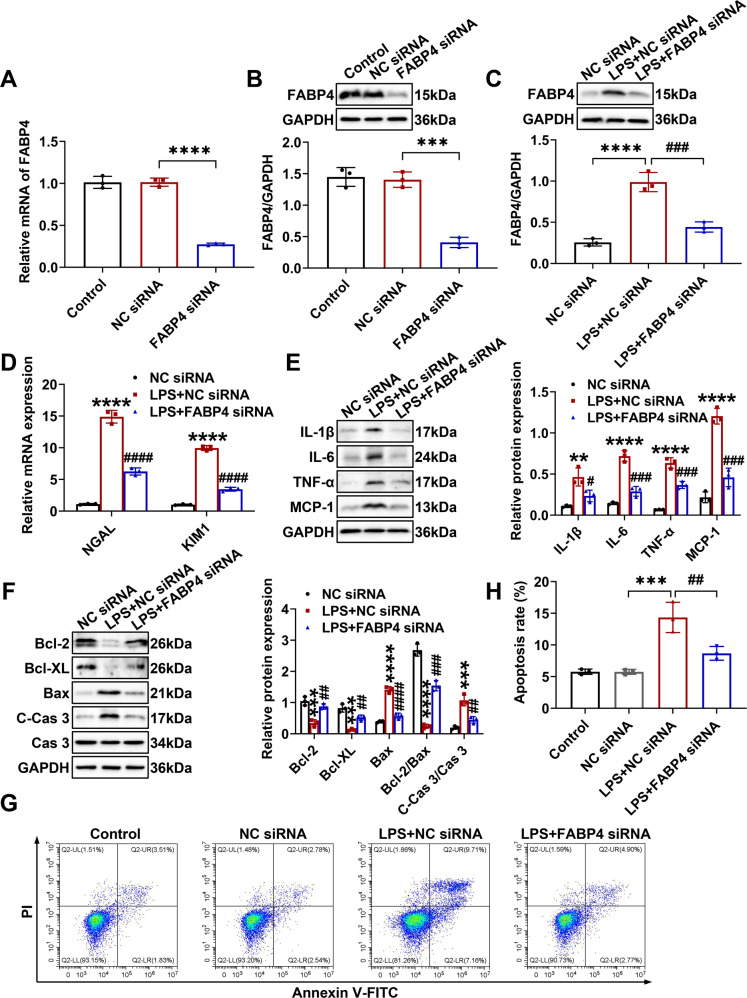
FABP4 knockdown inhibited inflammation and apoptosis in LPS-stimulated TCMK-1 cells. TCMK-1 cells were transfected with negative control (NC) siRNA or FABP4 siRNA for 24 h and then treated with 100 μg/ml LPS for another 24 h. The knockdown efficiency of FABP4 siRNA in TCMK-1 cells was evaluated by (**A**) quantitative real-time PCR analysis and (**B**) western blot analysis. **C** Representative western blot images and quantitative analysis of FABP4 in TCMK-1 cells. **D** The mRNA levels of NGAL and KIM1 detected by quantitative real-time PCR. Western blotting and densitometry quantification of (**E**) inflammatory factors including IL-1β, IL-6, TNF-α, and MCP-1, and (**F**) apoptotic marker including Bcl-2, Bcl-XL, Bax, caspase 3 (Cas 3), and cleaved-caspase 3 (C-Cas 3) in TCMK-1 cells. **G** Representative flow cytometric plots of TCMK-1 cell apoptosis and (**H**) quantification of apoptosis rate. All data are displayed as mean ± SD (*n* = 3); ^**^P < 0.01, ^***^P < 0.001^, ****^P < 0.0001 versus NC siRNA; ^#^*P* < 0.05, ^##^*P* < 0.01, ^###^*P* < 0.001, ^####^*P* < 0.0001 versus LPS + NC siRNA.

Next, TCMK-1 cells were transfected with FABP4 siRNA, which suppressed FABP4 expression (Fig. 5A, B). After LPS stimulation, TCMK-1 cells showed an increased FABP4 expression, whereas reversed by FABP4 siRNA (Fig. 5C). Further, FABP4 knockdown attenuated LPS-stimulated cellular injury, inflammation, and apoptosis, as evidenced by a reduction of NGAL, KIM1 mRNA levels (Fig. 5D), proinflammatory cytokines (IL-1β, IL-6, TNF-α, and MCP-1) (Fig. 5E), pro-apoptotic markers (cleaved-caspase 3 and Bax) (Fig. 5F) and apoptotic rate by flow cytometry (Fig. 5G, H), as well as an increase of anti-apoptotic markers (Bcl-2 and Bcl-XL) (Fig. 5F).

**Fig. 6:**
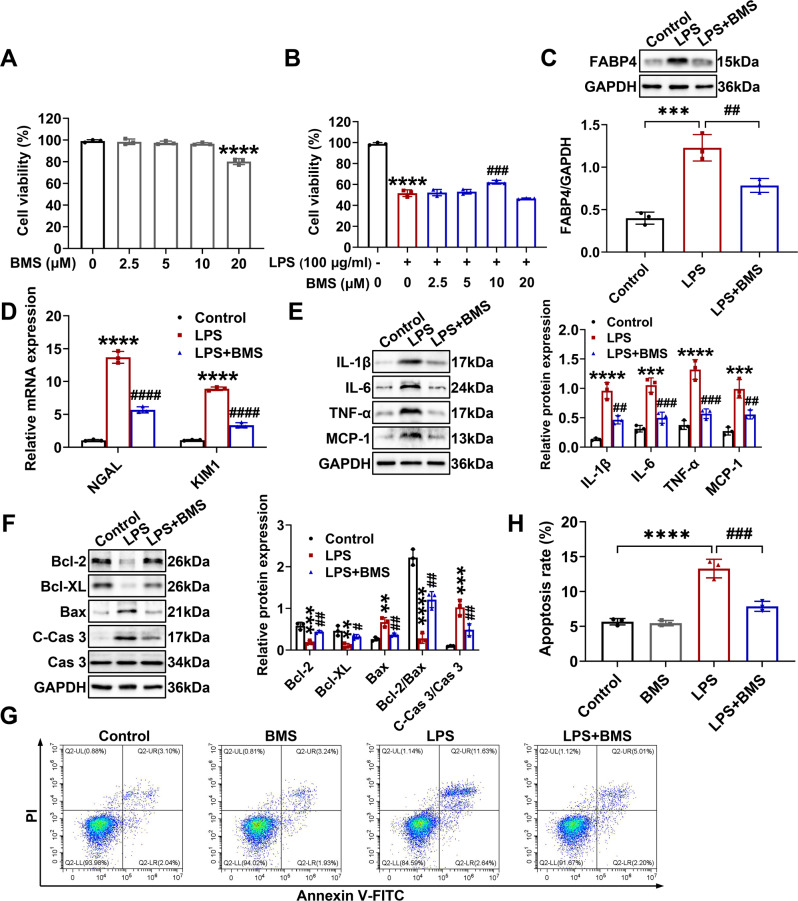
FABP4 inhibitor BMS309403 treatment suppressed inflammation and apoptosis in LPS-stimulated TCMK-1 cells. TCMK-1 cells were either untreated or pretreated with BMS309403 (BMS) for 30 min before 24 h of 100 μg/ml LPS stimulation. **A** The cytotoxic effect of BMS (0, 2.5, 5, 10, and 20 μM) on TCMK-1 cells for 24 h determined by Cell Counting Kit-8 assay (mean ± SD, *n* = 3). ^****^P < 0.0001 versus BMS (μM) 0 group. **B** The cytoprotective effect of BMS (0, 2.5, 5, 10, and 20 μM) on LPS-stimulated TCMK-1 cells for 24 h determined by Cell Counting Kit-8 assay (mean ± SD, *n* = 3). ^****^P < 0.0001 versus BMS (μM) 0 with LPS (100 μg/ml) - group; ^###^*P* < 0.001 versus BMS ^(μ^M) 0 with LPS (100 μg/ml) + group. **C**–**H** 10 μM BMS was chosen to treat LPS-stimulated TCMK-1 cells. **C** Western blotting and quantification by densitometry of FABP4 in TCMK-1 cells. **D** Quantitative real-time PCR analysis of NGAL and KIM1 in TCMK-1 cells. Representative western blots and densitometry quantification of (**E**) inflammatory factors including IL-1β, IL-6, TNF-α, and MCP-1, and (**F**) apoptotic marker including Bcl-2, Bcl-XL, Bax, caspase 3 (Cas 3), and cleaved-caspase 3 (C-Cas 3) in TCMK-1 cells. **G** Representative flow cytometric plots of TCMK-1 cell apoptosis and (**H**) quantification of apoptosis rate. Data are shown as mean ± SD (*n* = 3); ^**^P < 0.01, ^***^P < 0.001, ^****^P ^<^ 0.0001 versus Control^; #^*P* < 0.05, ^##^*P* < 0.01, ^###^*P* < 0.001, ^####^*P* < 0.0001 versus LPS.

Furthermore, we used CCK-8 assay to examine the potential cytotoxic effect of BMS309403 (0-20 μM) in TCMK-1 cells and found that when the concentration of BMS309403 was elevated to 20 μM, the cell viability of TCMK-1 cells would be obviously inhibited at 24 h (Fig. 6A). Although as low as 2.5 μM inhibitor was enough to suppress the mRNA expression of FABP4 in TCMK-1 cells (Fig. S6), only 10 μM BMS309403 significantly restored cell viability in LPS-stimulated TCMK-1 cells (Fig. 6B). Consequently, 10 μM BMS309403 was ultimately selected for experiments. As illustrated in Fig. 6C, LPS-induced FABP4 expression was suppressed by BMS309403 in TCMK-1 cells. BMS309403 also obtained remarkable cytoprotective (Fig. 6D), anti-inflammatory (Fig. 6E), and anti-apoptotic (Fig. 6F–H) effect in LPS-stimulated TCMK-1 cells. Overall, the above results indicated that FABP4 was induced in TCMK-1 cells by LPS, while genetic and pharmacologic inhibition of FABP4 protected TCMK-1 cells against LPS by inhibiting inflammation and apoptosis.

### Upregulation of FABP4 was dependent on TLR4/c-Jun signaling activation in LPS-stimulated TCMK-1 cells

**Fig. 7:**
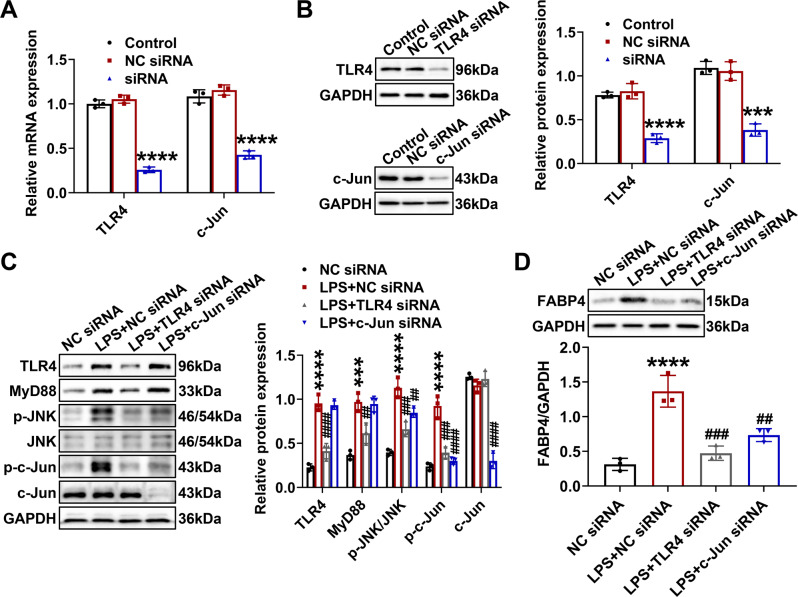
Upregulation of FABP4 was mediated by TLR4/c-Jun signaling activation in LPS-stimulated TCMK-1 cells. TCMK-1 cells were transfected with negative control (NC) siRNA, TLR4 siRNA, or c-Jun siRNA for 24 h and then treated with 100 μg/ml LPS for another 24 h. The knockdown efficiencies of TLR4 siRNA and c-Jun siRNA in TCMK-1 cells were evaluated by (**A**) quantitative real-time PCR analysis and (**B**) western blot analysis. **C** Western blotting and densitometry quantification of TLR4, MyD88, p-JNK, JNK, p-c-Jun, and c-Jun in TCMK-1 cells. **D** FABP4 protein expression detected by western blotting and quantified by densitometry in TCMK-1 cells. All data are represented as mean ± SD (*n* = 3); ^***^P < 0.001, ^****^P < 0.0001 versus NC siRNA; ^##^*P* < 0.01, ^###^*P* < 0.001, ^####^*P* < 0.0001 versus LPS + NC siRNA.

As presented in Fig. 7A, B, TCMK-1 cells were separately transfected with TLR4 siRNA and c-Jun siRNA, which suppressed the expression of TLR4 and c-Jun, respectively. Compared with those of LPS-untreated cells, the expression of TLR4, MyD88, p-JNK, and p-c-Jun of LPS-stimulated cells exhibited higher levels (Fig. 7C). And these corresponding results were reversed by TLR4 siRNA, while c-Jun siRNA inhibited the levels of c-Jun and p-c-Jun without influencing the expression of TLR4 and MyD88 in LPS-stimulated TCMK-1 cells (Fig. 7C). Interestingly, the level of p-JNK from LPS-stimulated cells was also suppressed by c-Jun siRNA (Fig. 7C), while c-Jun is a direct substrate of JNK [29], and this result implied that there might be a positive feedback loop between c-Jun and JNK in LPS-stimulated TCMK-1 cells.

Afterwards, TLR4 siRNA and c-Jun siRNA both alleviated the inflammatory responses (Fig. S7A) and apoptosis (Fig. S7B–D) in LPS-stimulated TCMK-1 cells. Most importantly in Fig. 7D, the LPS-induced FABP4 expression was largely inhibited by transfection of TLR4 siRNA or c-Jun siRNA in TCMK-1 cells. Collectively, LPS-induced upregulation of FABP4 was dependent on the activation of TLR4/c-Jun signaling in TCMK-1 cells.

### FABP4 mediated c-Jun activation via a positive feedback loop in septic AKI

**Fig. 8:**
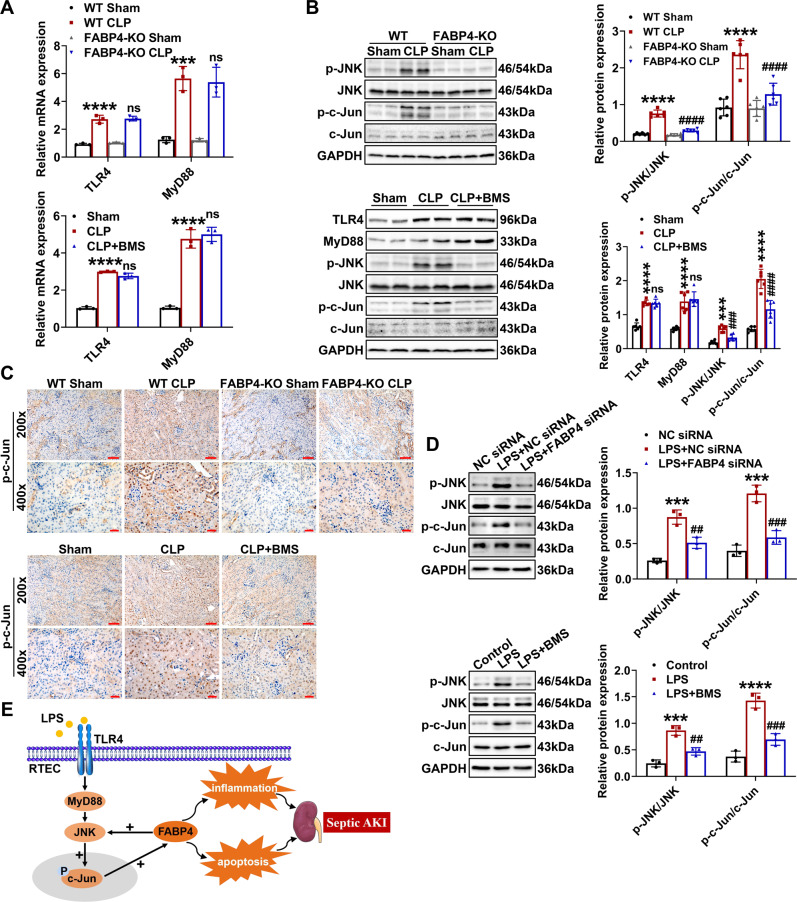
Tubular FABP4 formed a positive feedback loop with c-Jun in septic AKI. C57BL/6J, FABP4 inhibitor BMS309403 (BMS)-treated C57BL/6J, FABP4 KO, and WT mice (male, 8–10 weeks old) were subjected to CLP or sham surgery and killed 16 h later. TCMK-1 cells were transfected with negative control (NC) siRNA or FABP4 siRNA for 24 h and then treated with 100 μg/ml LPS for another 24 h, or either untreated or pretreated with 10 μM BMS for 30 min before 24 h of 100 μg/ml LPS stimulation. **A** Renal mRNA levels of TLR4 and MyD88 measured by quantitative real-time PCR (*n* = 3). **B** Renal protein levels of TLR4, MyD88, p-JNK, JNK, p-c-Jun, and c-Jun examined by western blotting and quantified by densitometry (*n* = 6). **C** Immunochemistry staining of p-c-Jun in kidney tissue sections (×200, scale bar = 50 μm; ×400, scale bar = 20 μm). Data are shown as mean ± SD; ^***^P < 0.001, ^****^P < 0.0001 for WT CLP versus WT Sham, or for CLP versus Sham; ns = no significance, ^###^*P* < 0.001, ^####^*P* < 0.0001 for FABP4-KO CLP versus WT CLP, or for CLP + BMS versus CLP. **D** Western blotting and densitometry quantification of p-JNK, JNK, p-c-Jun, and c-Jun in TCMK-1 cells. Data are shown as mean ± SD (*n* = 3); ^***^P < 0.001, ^****^P < 0.0001 for LPS + NC siRNA versus NC siRNA, or for LPS versus Control; ^##^*P* < 0.01, ^###^*P* < 0.001 for LPS + FABP siRNA versus LPS + NC siRNA, or for LPS + BMS versus LPS. **E** Schematic illustration of the role and regulation of tubular FABP4 in the pathogenesis of septic AKI.

Given that FABP4 was reported to modulate TLR4 signaling and JNK/c-Jun cascade in different pathological processes [27, 30], and combined with our findings, we speculated that a positive feedback loop might exist between FABP4 and TLR4/MyD88/JNK/c-Jun signaling, which further amplifies inflammatory response and cell apoptosis in septic AKI. As indicated by RT-qPCR and western blotting, we observed that FABP4 inhibition by genetic deletion or BMS309403 treatment significantly suppressed CLP-induced expression of p-JNK and p-c-Jun without changing TLR4 and MyD88 levels (Fig. 8A, B). As further confirmed by immunochemistry results, the CLP-induced p-c-Jun which was mainly located in the nucleus of RTECs was markedly reduced by FABP4 deficiency or BMS309403 (Fig. 8C). Consistently, LPS-induced expression of p-JNK and p-c-Jun were suppressed by FABP4 siRNA or BMS309403 in TCMK-1 cells (Fig. 8D). Altogether, tubular FABP4 formed a positive feedback loop with c-Jun in septic AKI, and this regulatory relationship might be achieved by JNK activation (Fig. 8E).

### RTEC-specific deletion of FABP4 mitigated CLP-induced septic AKI

**Fig. 9:**
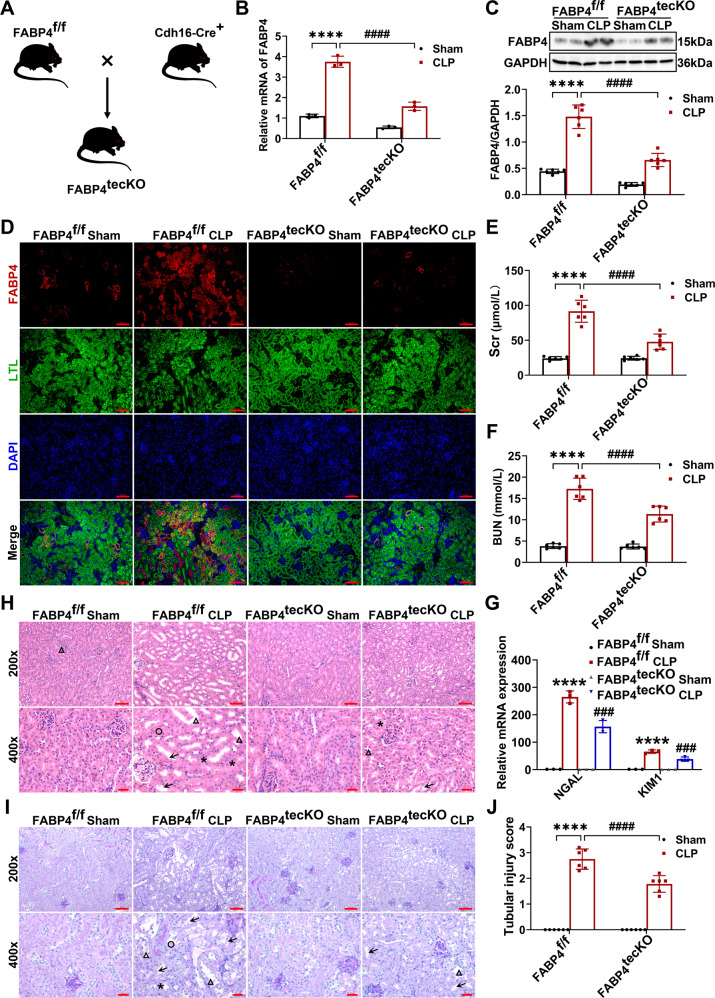
Renal tubular epithelial cell-specific (RTEC-specific) deletion of FABP4 attenuated CLP-induced septic AKI in mice. RTEC-specific FABP4 KO (FABP4^tecKO^) and FABP4^flox/flox^ (FABP4^f/f^) mice (male, 8–10 weeks old) were subjected to CLP or sham surgery and killed 16 h later. **A** Mating strategy to generate FABP4 conditional KO in mouse RTECs. **B** FABP4 mRNA expression in renal cortex measured by quantitative real-time PCR (*n* = 3). **C** Western blot analysis of FABP4 protein expression in renal cortex (*n* = 6). **D** Representative micrographs of immunofluorescence staining of FABP4 (red) and proximal RTEC marker LTL (green) in renal cortex (×200, scale bar = 50 μm). **E** Serum creatinine (Scr) and (**F**) BUN levels in different groups of mice (*n* = 6). **G** Renal NGAL and KIM1 mRNA expression measured by quantitative real-time PCR (*n* = 3). Representative (**H**) HE and (**I**) PAS staining micrographs (×200, scale bar = 50 μm; ×400, scale bar = 20 μm) and (**J**) tubular injury scores of kidney tissues based on HE staining (*n* = 6). Triangle: tubular dilatation; Asterisk: tubular swelling; Circle: cast formation; Arrow: loss of brush border. All data are displayed as mean ± SD; ^****^P < 0.0001, versus WT FABP4^f/f^ Sham; ^###^*P* < 0.001^, ####^*P* < 0.0001, versus FABP4^f/f^ CLP.

Finally, to assess the role of RTEC-specific FABP4 in vivo, we firstly constructed FABP4^flox/flox^ (FABP4^f/f^) mice by CRISPR/Cas9-stimulated homologous recombination (Fig. S1A). Then we crossed FABP4^f/f^ mice with RTEC-specific cadherin-16 (Cdh16)-Cre transgenic mice (Fig. 9A). Mice with RTEC-specific ablation of FABP4 (Cdh16-Cre^+^ FABP4^f/f^, here referred to as FABP4^tecKO^) were confirmed by PCR assay (Fig. S1B), whereas age- and gender-matched FABP4^f/f^ littermates were considered as controls.

To determine FABP4 expression in kidney cortices from FABP4^tecKO^ and FABP4^f/f^ mice, western blotting, RT-qPCR, and immunofluorescent staining were used. The mRNA and protein levels of FABP4 were inhibited in kidneys of FABP4^tecKO^ mice compared with those of FABP4^f/f^ mice (Fig. 9B, C). Meanwhile, FABP4 was mainly upregulated in proximal tubular epithelial cells (marked by LTL) in FABP4^f/f^ mice after CLP, while reduced in most of the proximal tubules in FABP4^tecKO^ mice (Fig. 9D), indicating an effective deletion of FABP4 in RTECs. Moreover, no obvious differences in renal function, tubular injury and histopathologic consequences were found between FABP4^tecKO^ sham and FABP4^f/f^ sham mice (Fig. 9E–J).

As displayed in Fig. 9E–G, the Scr, BUN, and kidney mRNA levels of NGAL and KIM1 were remarkably increased in FABP4^f/f^ mice subjected to CLP, while significantly inhibited in FABP4^tecKO^ mice. The kidney histology analysis also obtained similar data by PAS and HE stainings (Fig. 9H–J). In summary, these findings suggested that RTEC-specific FABP4 deletion mitigated septic AKI induced by CLP.

## Discussion

Although efforts have been made to explore the underlying mechanism of septic AKI, no specific treatment has been proven effective. In the study, we presented the following findings: (a) FABP4 was markedly upregulated in RTECs in experimental models of septic AKI; (b) functionally, inhibition of FABP4 attenuated sepsis-induced kidney injury, and particularly, RTEC-specific deletion of FABP4 also showed similar renoprotective effects; (c) mechanistically, upregulation of tubular FABP4 in septic AKI was mediated by TLR4/c-Jun signaling activation, and FABP4 formed a positive feedback loop with c-Jun to exacerbate kidney inflammation and apoptosis. Together, these findings provided a novel insight into the role of tubular FABP4 in septic AKI.

Increasing evidence suggests that inflammatory response and cell apoptosis play critical roles in septic AKI [5, 28, 31]. In the present study, we initially found that tubular FABP4 was induced by sepsis, and inhibition of FABP4 by genetic deletion or BMS309403 treatment both ameliorated impaired renal function and alleviated tubular damage through suppressing inflammation and cell apoptosis in septic AKI mice. These findings revealed that FABP4 played important roles in septic AKI pathogenesis by regulating inflammation and cell apoptosis. Corroborating with our results, recent studies also showed that FABP4 inhibition could suppress saturated-fatty-acid-induced skeletal muscle inflammation and preeclampsia inflammasome activation [32, 33].

TLR4, a pattern recognition receptor, is constitutively expressed in RTECs, and involves inflammation and cell apoptosis in septic AKI [12, 13, 34]. In this study, we also found that TLR4 was upregulated in RTECs by CLP, while TLR4 deficiency or TAK242 administration both inhibited the elevation of TLR4 and FABP4 in renal tubules of CLP-treated mice. Meanwhile, as anticipated, kidney damage was ameliorated, and the increased kidney inflammation and apoptosis were also reduced by TLR4 inhibition in CLP-treated mice. Collectively, these data suggested that CLP-induced upregulation of tubular FABP4 was mediated by TLR4 signaling activation.

As a specific exogenous ligand of TLR4, LPS serves as endotoxins and immunogens, and is widely used to establish septic AKI experimental model [7, 10]. Consistent with our in vivo results, TLR4 and FABP4 were induced in LPS-stimulated TCMK-1 cells, while TLR4 siRNA inhibited the elevated levels of TLR4 and FABP4, and the production of proinflammatory cytokines and cell apoptosis were also reduced. Taken together, our data strongly suggested that induction of tubular FABP4 in septic AKI was dependent on TLR4 signaling activation. As a key molecule to transduce signals from TLR4 to downstream pathways, MyD88 also has crucial roles in sepsis-induced organ injuries [14, 35, 36]. Here, in our mouse and TCMK-1 cell models of septic AKI, a significant increase in the level of MyD88 was suppressed by TLR4 inhibition, and the elevation of MyD88 might take part in the induction of FABP4, while the precise relationship still needs to be further confirmed.

In parallel, we found that the activation of JNK/c-Jun cascade in CLP-injured kidneys and LPS-stimulated TCMK-1 cells was repressed by TLR4 inhibition. c-Jun is a subunit of AP-1 transcription factor, and specifically, the activated c-Jun via its phosphorylation forms AP-1 complex, which then regulates different genes transcription involved in inflammation, apoptosis, and proliferation [37–39]. In addition, JNK is the key kinase of c-Jun, while c-Jun is also the major downstream target of JNK, as the functions of JNK depend mainly on phosphorylating c-Jun [29, 39]. Accumulating evidence indicates that JNK/c-Jun cascade plays a pronounced role in various renal pathological situations such as kidney inflammation, tubular apoptosis, and tubulointerstitial fibrosis [15, 17, 40]. In our study, knockdown of c-Jun inhibited inflammation and apoptosis in LPS-stimulated TCMK-1 cells, and more importantly, the expression of FABP4 was also suppressed. Overall, these data suggested that the upregulation of tubular FABP4 in septic AKI was mediated by TLR4/c-Jun signaling activation.

Interestingly, in the current study, FABP4 inhibition suppressed JNK/c-Jun cascade activation in CLP-injured kidneys and LPS-stimulated TCMK-1 cells, while the levels of TLR4 and MyD88 in CLP-injured kidneys were not influenced by BMS309403 treatment. As such, tubular FABP4 could in turn mediate the activation of c-Jun in septic AKI, likely by activating JNK. Eventually, FABP4 may form a positive feedback loop with c-Jun to amplify inflammation and apoptosis in RTECs of septic AKI (Fig. 8E).

As noted, despite considerable work about FABP4 in several forms of AKI in our previous studies was performed [25–27], there is still a lack of knowledge concerning the mechanism leading to FABP4 upregulation. In the present study, we reported for the first time that TLR4/c-Jun signaling was essential for tubular FABP4 upregulation in septic AKI. Furthermore, the positive feedback loop between FABP4 and c-Jun was found in septic AKI in this study, and thus provided a rational theoretical basis for how FABP4 modulated inflammation and apoptosis in RTECs and consequently aggravated septic AKI. Although by using FABP4 KO mice, previous studies suggested that FABP4 may play a role in several diseases such as cisplatin-induced AKI, cerebral ischemia injury, and osteoarthritis [25, 30, 41], while the cell type responsible for disease pathogenesis was difficult to clarify. Notably, previous studies have reported that FABP4 can be ectopically expressed in injured endothelial cells in the glomerulus and arterial endothelial cells of the hyperplastic neointima [42, 43]. In the current study, in CLP-injured kidneys, we found that there was no expression of FABP4 in glomerular endothelial cells, and a certain expression of FABP4 in renal interstitial vascular endothelial cells, and a marked expression of FABP4 in RTECs (Fig. 2C and 9D). The above difference in FABP4 expression patterns may be due to different disease models. By taking the lead in using RTEC-specific FABP4 KO mice, we demonstrated a direct role of tubular FABP4 in septic AKI.

Although we elucidated a critical role of tubular FABP4 in sepsis-induced kidney injury and its inhibition might be a potential strategy for treating septic AKI, there still exist several limitations to our findings. Firstly, prophylactic administration of BMS309403 and TAK242 in vivo were performed before modeling. Although this administration mode was broadly adopted in previous studies [26, 44], future studies should consider different ways of administration that are more amenable to use in clinic. Secondly, informed by the work reported here, further investigation is needed to complete our understanding of how FABP4 regulates JNK/c-Jun cascade activation in septic AKI.

In conclusion, our study illustrated that genetic or pharmacologic inhibition of FABP4 alleviated septic AKI. Mechanistically, upregulation of tubular FABP4 in septic AKI was mediated by TLR4/c-Jun signaling activation and FABP4 formed a positive feedback loop with c-Jun to enhance inflammation and apoptosis. Therefore, FABP4 may be a therapeutic target for septic AKI.

## Supplementary information

Raw Western Blot Images

Supplementary Materials

Reproducibility Checklist

## Data Availability

All data supporting this research has been included in this manuscript and its supplementary information files. Raw sequencing data for this study are available in the NCBI SRA repository under accession number SRP356238.
