# Supplementary material for: Fatty acid-binding protein 4 is a therapeutic target for septic acute kidney injury by regulating inflammatory response and cell apoptosis
Source: Cell Death Dis. 2022 Apr 11;13(4):333. doi: 10.1038/s41419-022-04794-w (PMC9001746; doi:10.1038/s41419-022-04794-w)

Raw Western Blot Images

This PDF file includes the raw images for western blot presented in this paper.

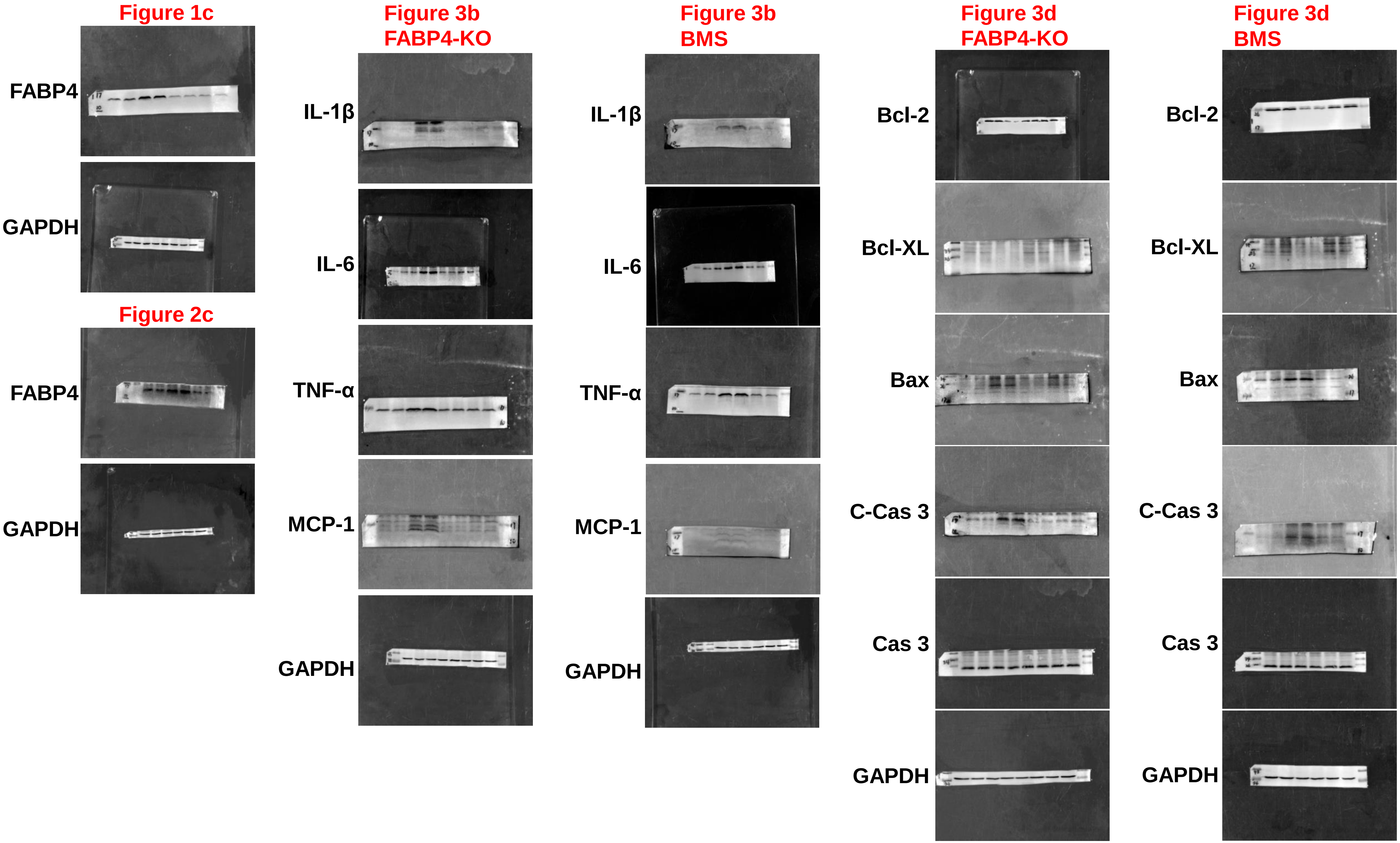
Figure 1c
FABP4
GAPDH
Figure 2c
FABP4
GAPDH
Figure 3b FABP4-KO
IL-1β
IL-6
TNF-α
MCP-1
GAPDH
Figure 3b BMS
IL-1β
IL-6
TNF-α
MCP-1
GAPDH
Figure 3d FABP4-KO
Bcl-2
Bcl-XL
Bax
C-Cas 3
Cas 3
GAPDH
Figure 3d BMS
Bcl-2
Bcl-XL
Bax
C-Cas 3
Cas 3
GAPDH

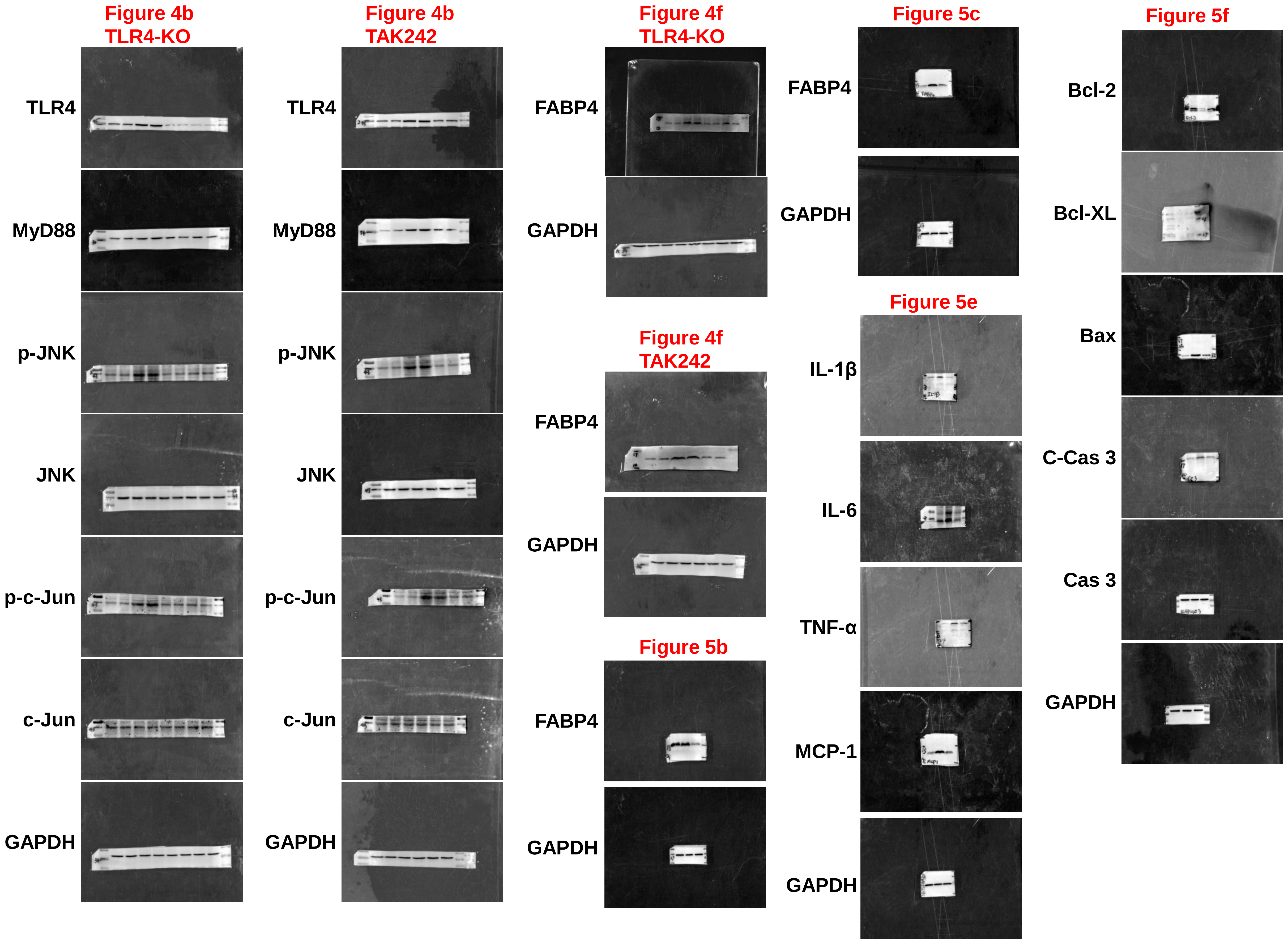
Figure 4b
TLR4-KO
TLR4
MyD88
p-JNK
JNK
p-c-Jun
c-Jun
GAPDH
Figure 4b
TAK242
TLR4
MyD88
p-JNK
JNK
p-c-Jun
c-Jun
GAPDH
Figure 4f
TLR4-KO
FABP4
GAPDH
Figure 4f
TAK242
FABP4
GAPDH
Figure 5b
FABP4
GAPDH
Figure 5c
FABP4
GAPDH
Figure 5e
IL-1β
IL-6
TNF-α
MCP-1
GAPDH
Figure 5f
Bcl-2
Bcl-XL
Bax
C-Cas 3
Cas 3
GAPDH

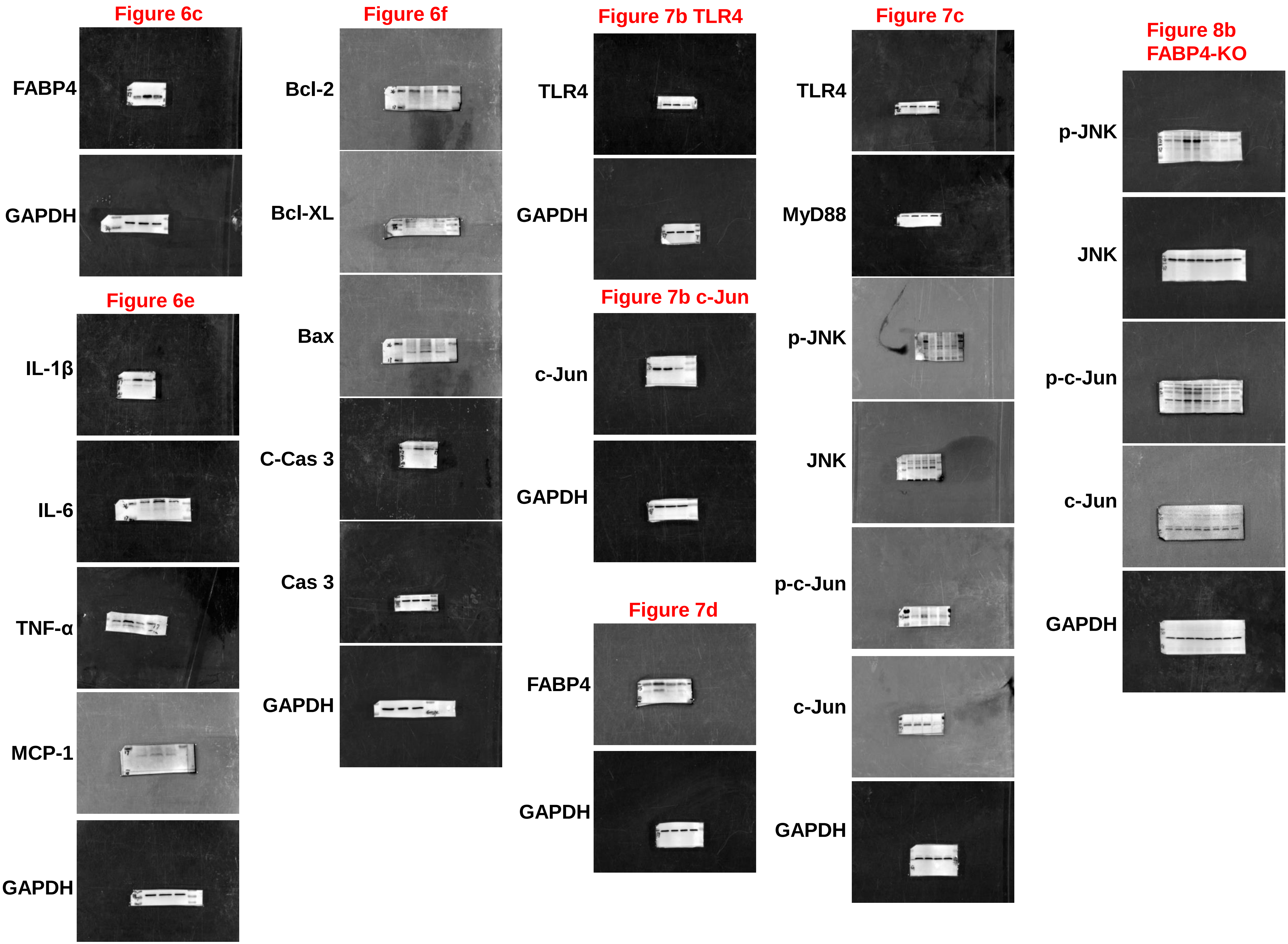
Figure 6c
FABP4
GAPDH
Figure 6e
IL-1β
IL-6
TNF-α
MCP-1
GAPDH
Figure 6f
Bcl-2
Bcl-XL
Bax
C-Cas 3
Cas 3
GAPDH
Figure 7b TLR4
TLR4
GAPDH
Figure 7b c-Jun
c-Jun
GAPDH
Figure 7d
FABP4
GAPDH
Figure 7c
TLR4
MyD88
p-JNK
JNK
p-c-Jun
c-Jun
GAPDH
Figure 8b
FABP4-KO
p-JNK
JNK
p-c-Jun
c-Jun
GAPDH

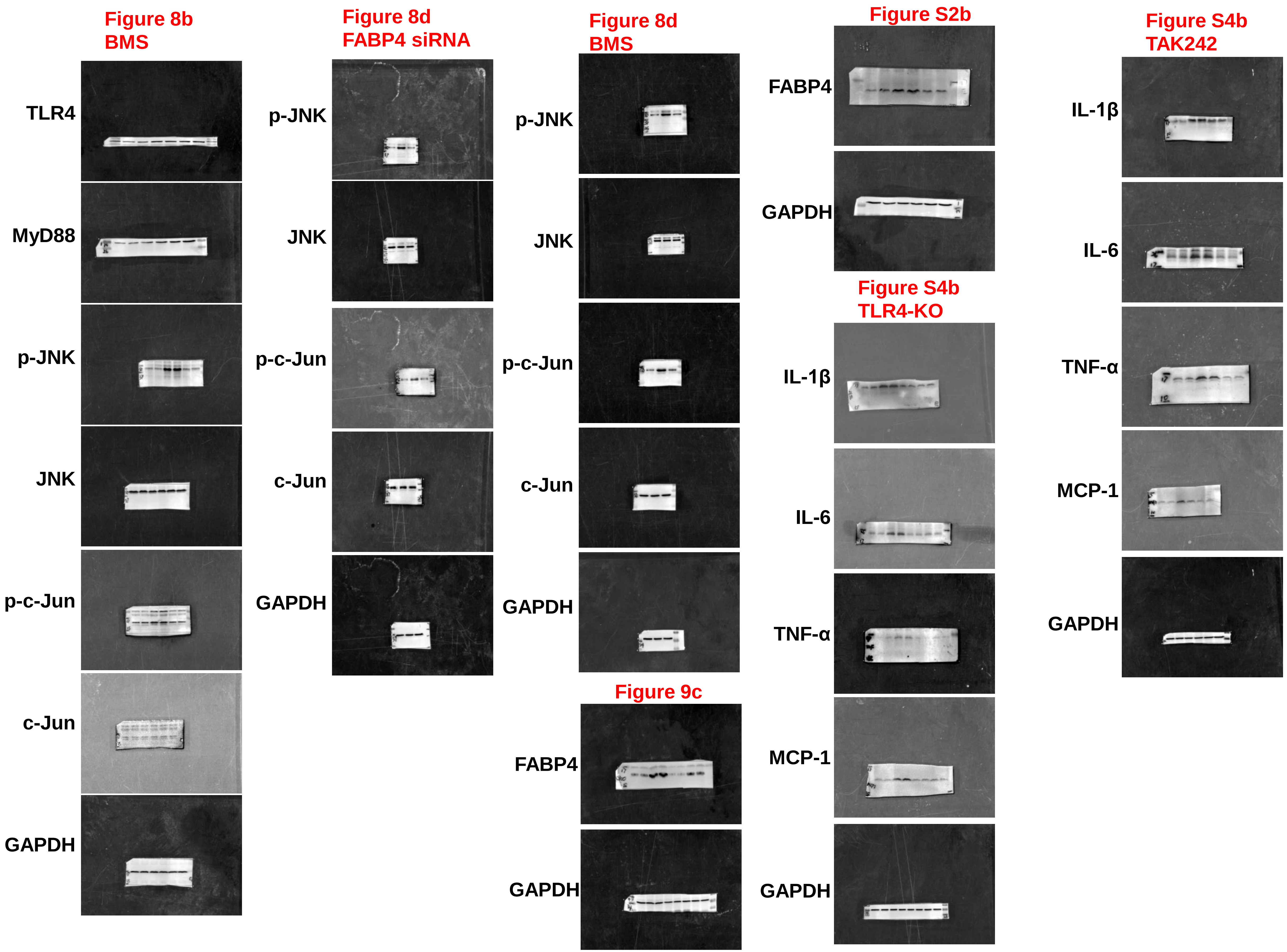

Figure 8b
BMS
TLR4
MyD88
p-JNK
JNK
p-c-Jun
c-Jun
GAPDH
Figure 8d
FABP4 siRNA
p-JNK
JNK
p-c-Jun
c-Jun
GAPDH
Figure 8d
BMS
p-JNK
JNK
p-c-Jun
c-Jun
GAPDH
Figure 9c
FABP4
GAPDH
Figure S2b
FABP4
GAPDH
Figure S4b
TLR4-KO
IL-1β
IL-6
TNF-α
MCP-1
GAPDH
Figure S4b
TAK242
IL-1β
IL-6
TNF-α
MCP-1
GAPDH

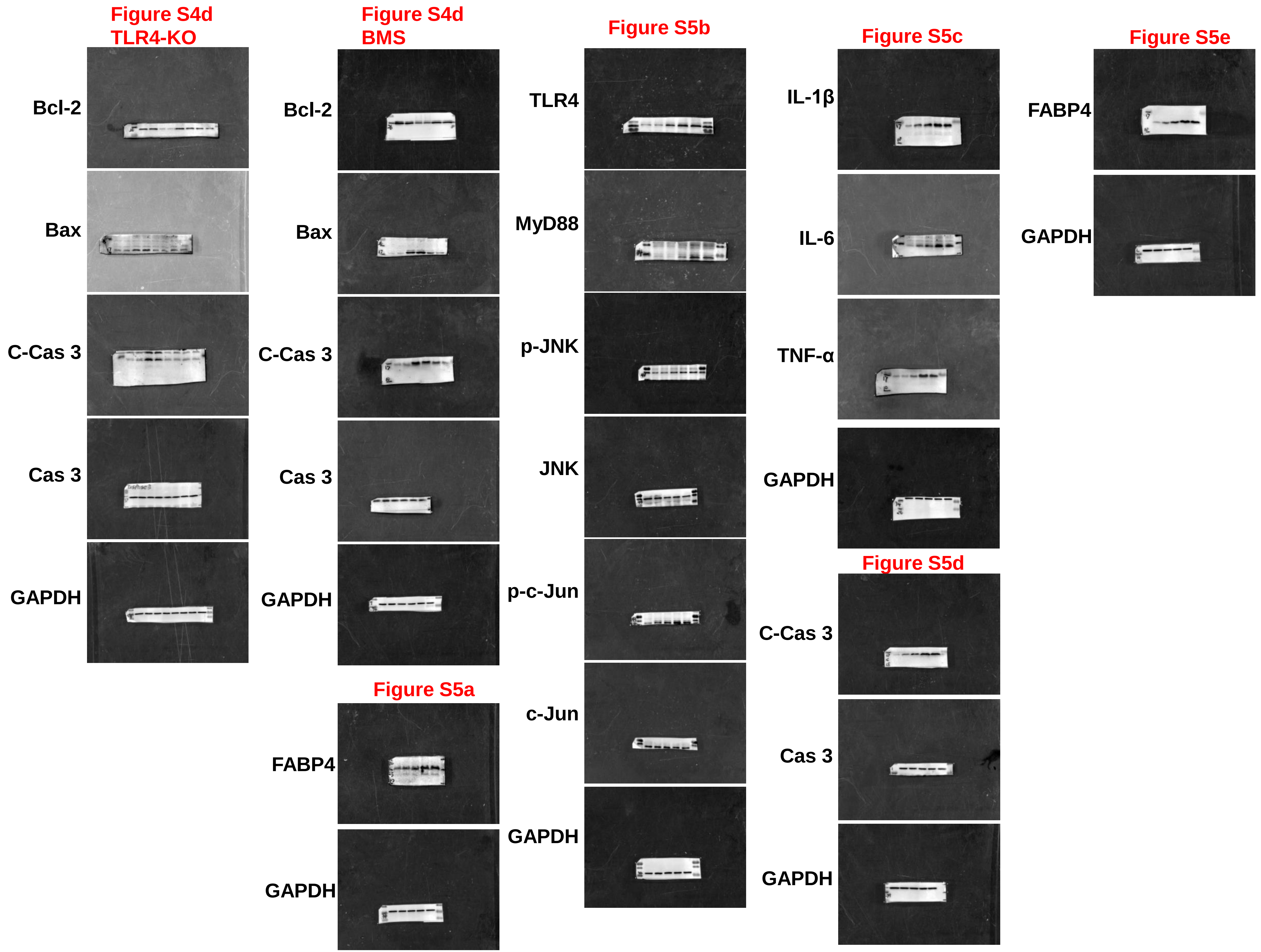

Figure S4d
TLR4-KO
Bcl-2
Bax
C-Cas 3
Cas 3
GAPDH
Figure S4d
BMS
Bcl-2
Bax
C-Cas 3
Cas 3
GAPDH
Figure S5a
FABP4
GAPDH
Figure S5b
TLR4
MyD88
p-JNK
JNK
p-c-Jun
c-Jun
GAPDH
Figure S5c
IL-1β
IL-6
TNF-α
GAPDH
Figure S5d
C-Cas 3
Cas 3
GAPDH
Figure S5e
FABP4
GAPDH

Figure S5f

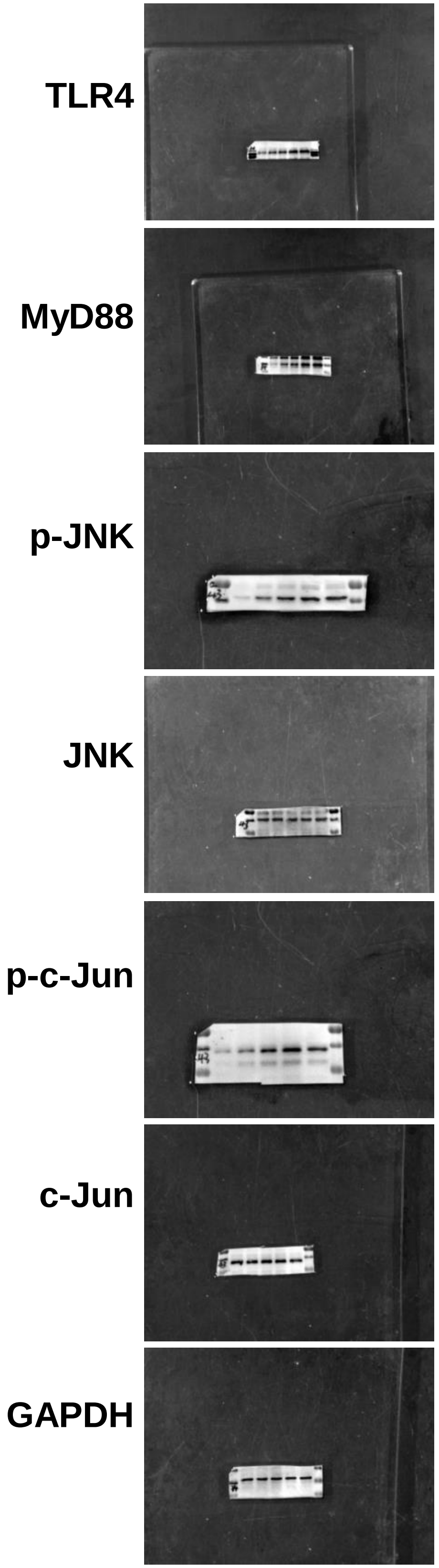

Figure S5g

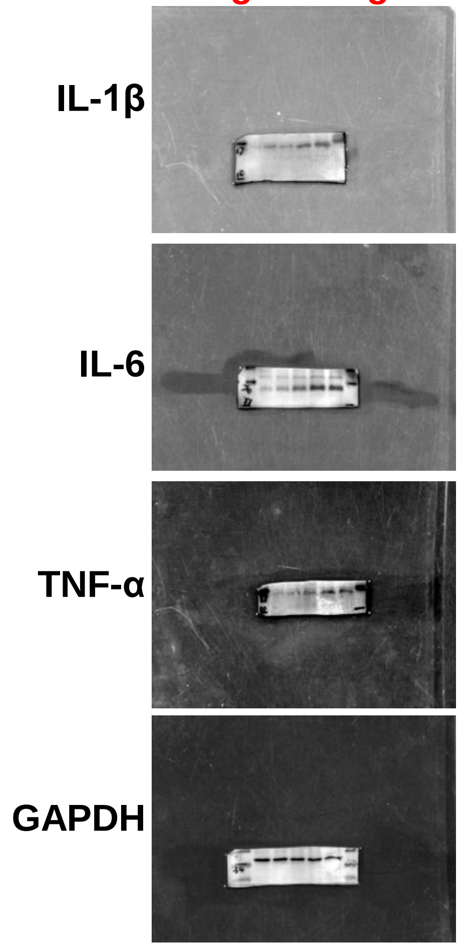

Figure S5h

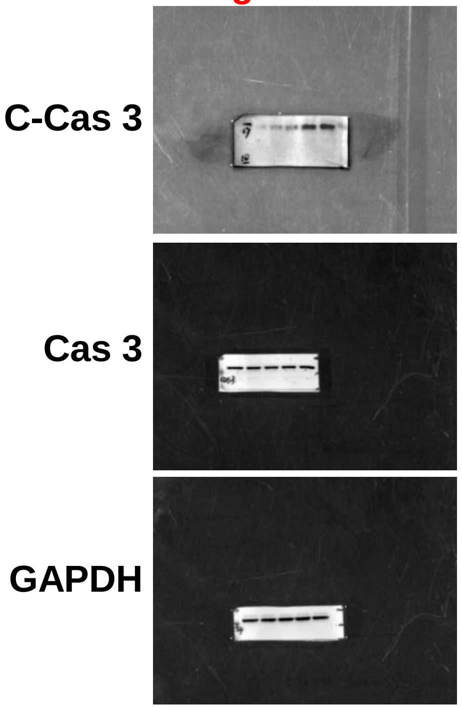

Figure S7a

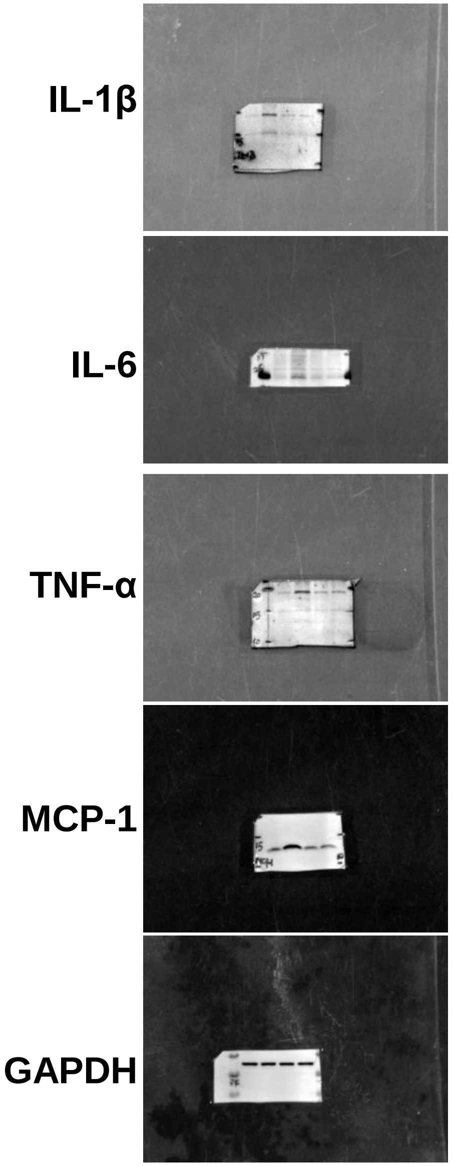

Figure S7b

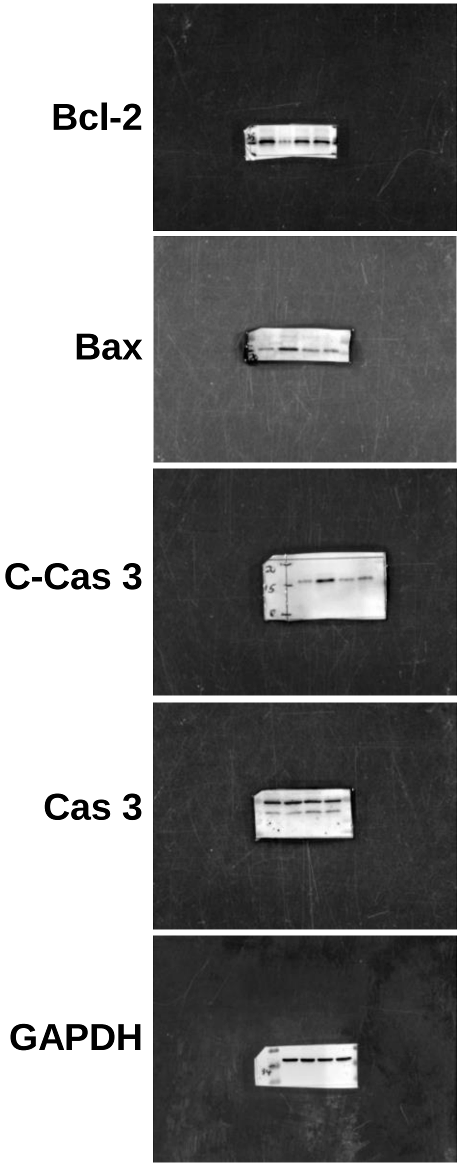

Supplement: Supplementary file 1 — Raw Western Blot Images [file 41419_2022_4794_MOESM1_ESM.pdf]
