# Supplementary material for: Fatty acid-binding protein 4 is a therapeutic target for septic acute kidney injury by regulating inflammatory response and cell apoptosis
Source: Cell Death Dis. 2022 Apr 11;13(4):333. doi: 10.1038/s41419-022-04794-w (PMC9001746; doi:10.1038/s41419-022-04794-w)
Supplement: Supplementary file 2 — Supplementary Materials [file 41419_2022_4794_MOESM2_ESM.docx]

**Supplementary Materials**

**Fatty acid-binding protein 4 is a therapeutic target for septic acute kidney injury by regulating inflammatory response and cell apoptosis**

Bo Wang ^1^, Jun Xu ^1^, Qian Ren ^1^, Lu Cheng ^1^, Fan Guo ^1^, Yan Liang ^2^, Letian Yang ^1^, Zhouke Tan ^3^, Ping Fu ^1,*^, Liang Ma^1,*^

^*^Corresponding authors.

Email addresses: Liang Ma ([Liang_m@scu.edu.cn](mailto:Liang_m@scu.edu.cn)) or Ping Fu ([fupinghx@scu.edu.cn](mailto:fupinghx@scu.edu.cn)).

**Supplementary Table of Contents**

1. **Supplementary Methods**

Animals and treatments and Cell siRNA transfection.

1. **Supplementary Figures**

**Fig. S1** Generation of renal tubular epithelial cell-specific (RTEC-specific) FABP4 KO mice.

**Fig. S2** FABP4 inhibitor BMS309403 treatment ameliorated LPS-induced septic AKI in mice.

**Fig. S3** TLR4 deficiency or TLR4 inhibitor TAK242 treatment attenuated CLP-induced septic AKI in mice.

**Fig. S4** TLR4 deficiency or TLR4 inhibitor TAK242 treatment alleviated CLP-induced kidney inflammation and apoptosis in mice.

**Fig. S5** Effect of doses and timing of LPS stimulation in TCMK-1 cells.

**Fig. S6** Effect of doses of FABP4 inhibitor BMS309403 treatment on FABP4 mRNA expression in TCMK-1 cells.

**Fig. S7** TLR4 or c-Jun knockdown inhibited inflammation and apoptosis in LPS-stimulated TCMK-1 cells.

1. **Supplementary Tables**

**Table S1.** Primary antibodies used in the experiments.

**Table S2.** Primer sequences used in real-time PCR analysis.

**1. Supplementary Methods**

**Animals and treatments**

8-10-week-old male C57BL/6J mice (25-27g) were obtained from Animal Laboratory Center of Sichuan University (Chengdu, China). FABP4 wild type (WT) and knockout (KO) mice in C57BL/6J background, FABP4^flox/flox^ (FABP4^f/f^) and renal tubular epithelial cell-specific (RTEC-specific) conditional FABP4 KO (Cdh16-Cre^+^ FABP4^f/f^, FABP4^tecKO^) mice in C57BL/6J background, TLR4 WT and KO mice in C57BL/10 background were purchased from Model Animal Research Center of Nanjing University (Nanjing, China). FABP4 KO target site and sequence details were previously described^1^. The construction of FABP4^f/f^ mice is based on CRISPR/Cas9-stimulated homologous recombination. Briefly, exon 2 and exon 3 of the FABP4 gene were flanked by two LoxP elements. Two heterozygous recombinant embryonic stem cells clones screened by homologous recombination were identified and microinjected into blastocysts from C57BL/6J mice to generate floxed heterozygous mice (FABP4^flox/+^). FABP4^flox/+^ mice were then inbred to obtain homozygous FABP4-floxed mice (FABP4^f/f^). To generate FABP4^tecKO^ mice, FABP4^f/f^ mice were crossed with Cdh16-Cre mice. The genotype of FABP4^tecKO^ mice was confirmed by PCR assay using specific primers (**Fig. S1**). Littermates carried the FABP4^f/f^ transgene were used as controls. All mice were housed in constant temperature (23±2 ℃) and a 12-h light/dark cycle condition with free access to food and water. The mice were adapted to the environment for 1 week before further research.

The mice (male, aged 8-10 weeks, 25-27g) were subjected to cecal ligation and puncture (CLP) or lipopolysaccharide (LPS）injection to induce septic AKI^2-4^. To establish CLP model, mice were anesthetized by intraperitoneal (i.p.) injection of pentobarbital (50mg/kg). After disinfecting the abdomen, a middle abdominal incision (1-2 cm) was performed to expose the cecum. The cecum was ligated at 1cm away from the blind end with a 4-0 silk suture and subsequently punctured twice using a 20-gauge needle. After gently squeezing the cecum, a small amount of feces was extruded from the perforation, the cecum was then returned to the abdominal cavity and the abdomen was closed using sterile suture in the end. Sham mice performed the same operation without ligation and puncture. All mice were resuscitated subcutaneously with 1ml prewarmed saline after surgery. To establish LPS model, LPS was administrated by a single i.p. injection at a dose of 10 mg/kg body weight, and control mice were injected with the same volume of 0.9% saline. FABP4 inhibitor BMS309403 was dissolved in 100ul 20% PEG400 and orally gavaged at a dose of 40 mg/kg/d for 3 consecutive days before CLP or LPS injection^1, 5^. TLR4 inhibitor TAK242 was dissolved in 100ul 0.9% DMSO and intraperitoneally injected at a dose of 3 mg/kg/d for 3 consecutive days before CLP^6^. The mice were euthanized (pentobarbital 50mg/kg, i.p.) and sacrificed at 16h after CLP or LPS injection, and kidney tissues and blood samples were collected and stored at -80℃ for various analysis.

**Cell siRNA transfection**

Transient transfections of the TCMK-1 cells with siRNAs were conducted with transfection reagent (ribo*FECT*^TM^ CP transfection kit (166T); RiboBio, Guangzhou, China) according to the manufacturer’s instructions. The sequences of FABP4 siRNA, TLR4 siRNA, c-Jun siRNA and negative control (NC) siRNA were as follows:

FABP4 siRNA, sense 5’-GUGGUGGAAUGUGUUAUGATT-3’ and antisense 5’-UCAUAACACAUUCCACCACTT-3’; TLR4 siRNA, sense 5’-GCUAUAGCUUCUCCAAUUUTT-3’ and antisense 5’-AAAUUGGAGAAGCUAUAGCTT-3’; c-Jun siRNA, sense 5’-GGCACAGCUUAAGCAGAAATT-3’ and antisense 5’-UUUCUGCUUAAGCUGUGCCTT-3’; NC siRNA sense 5’-UUCUCCGAACGUGUCACGUTT-3’ and antisense 5’-ACGUGACACGUUCGGAGAATT-3’ (GenePharma, Shanghai, China).

TCMK-1 cells were seeded in six-well plates and transfected with a final concentration of 100 nM siRNA using ribo*FECT*^TM^ CP reagent (RiboBio, Guangzhou, China) in MEM/EBSS medium (SH30024.01, Hyclone, Beijing, China) containing 0.5% fetal bovine serum (SH30084.03, HyClone, Beijing, China) without penicillinstreptomycin for 24 h. After transfection, TCMK-1 cells were treated with 100 μg/ml LPS for another 24 h.

**Supplementary References**

1. Tan Z, Guo F, Huang Z, Xia Z, Liu J, Tao S, et al. Pharmacological and genetic inhibition of fatty acid-binding protein 4 alleviated cisplatin-induced acute kidney injury. *J Cell Mol Med* **23**, 6260-6270 (2019).
2. Rittirsch D, Huber-Lang MS, Flierl MA, Ward PA. Immunodesign of experimental sepsis by cecal ligation and puncture. *Nat Protoc* **4**, 31-36 (2009).
3. Wu L, Gokden N, Mayeux PR. Evidence for the role of reactive nitrogen species in polymicrobial sepsis-induced renal peritubular capillary dysfunction and tubular injury. *J Am Soc Nephrol* **18**, 1807-1815 (2007).
4. Wang Y, Zhu J, Liu Z, Shu S, Fu Y, Liu Y, et al. The PINK1/PARK2/optineurin pathway of mitophagy is activated for protection in septic acute kidney injury. *Redox Biol* **38**, 101767 (2021).
5. Huang R, Shi M, Guo F, Feng Y, Feng Y, Liu J, et al. Pharmacological Inhibition of Fatty Acid-Binding Protein 4 (FABP4) Protects Against Rhabdomyolysis-Induced Acute Kidney Injury. *Front Pharmacol* **9**, 917 (2018).
6. Sha T, Sunamoto M, Kitazaki T, Sato J, Ii M, Iizawa Y. Therapeutic effects of TAK-242, a novel selective Toll-like receptor 4 signal transduction inhibitor, in mouse endotoxin shock model. *Eur J Pharmacol* **571**, 231-239 (2007).
7. **Supplementary Figures**


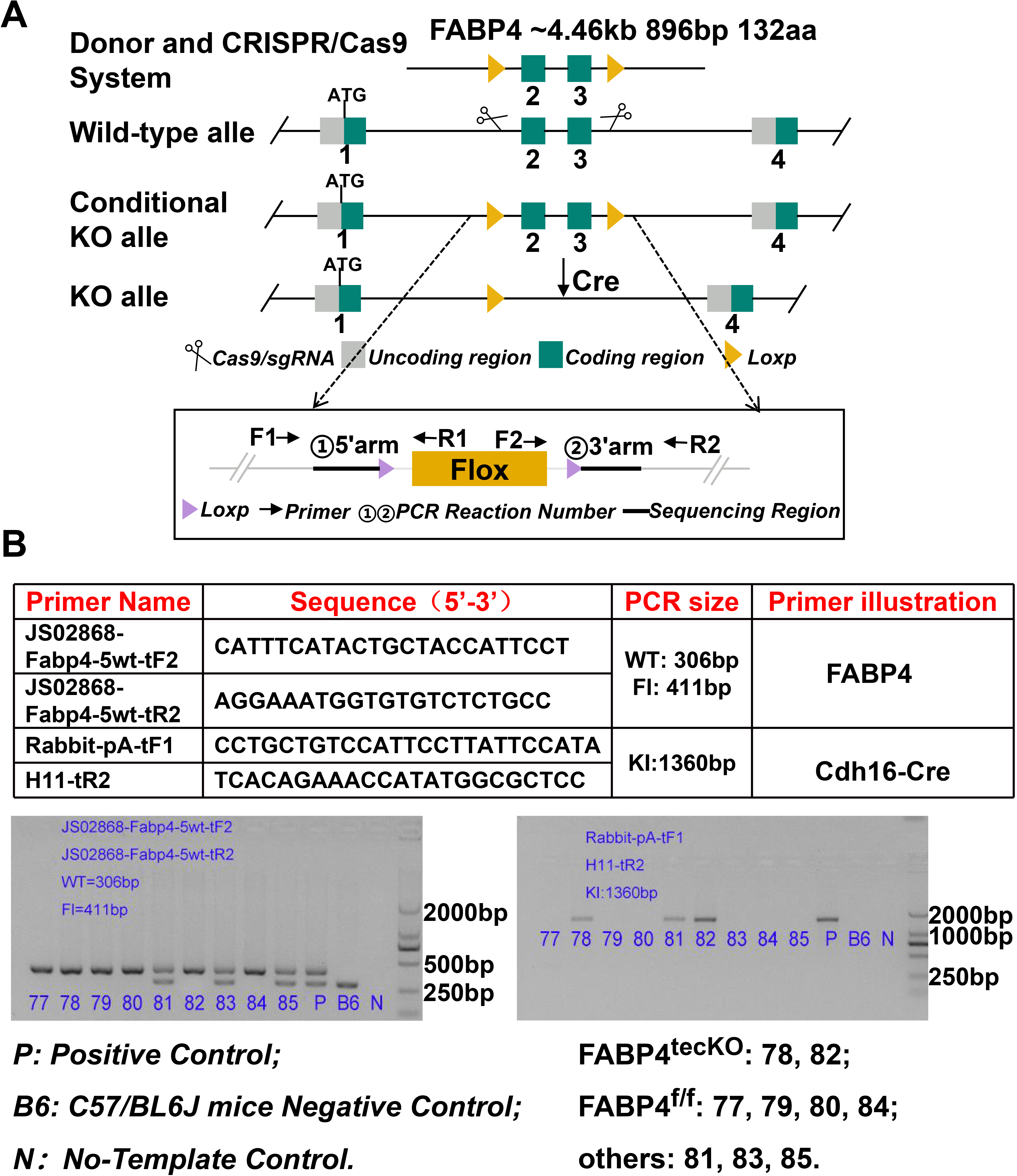


**Fig. S1 Generation of renal tubular epithelial cell-specific (RTEC-specific) FABP4 KO mice.** (**A**) Schematic of FABP4^flox/flox^ (FABP4^f/f^) mice generation by CRISPR/Cas9-stimulated homologous recombination and design strategy of RTEC-specific FABP4 KO (FABP4^tecKO^) mice. (**B**) identification of the genotype of FABP4^f/f^ mice and FABP4^tecKO^ (Cdh16-Cre^+^ FABP4^f/f^) mice by PCR assay.


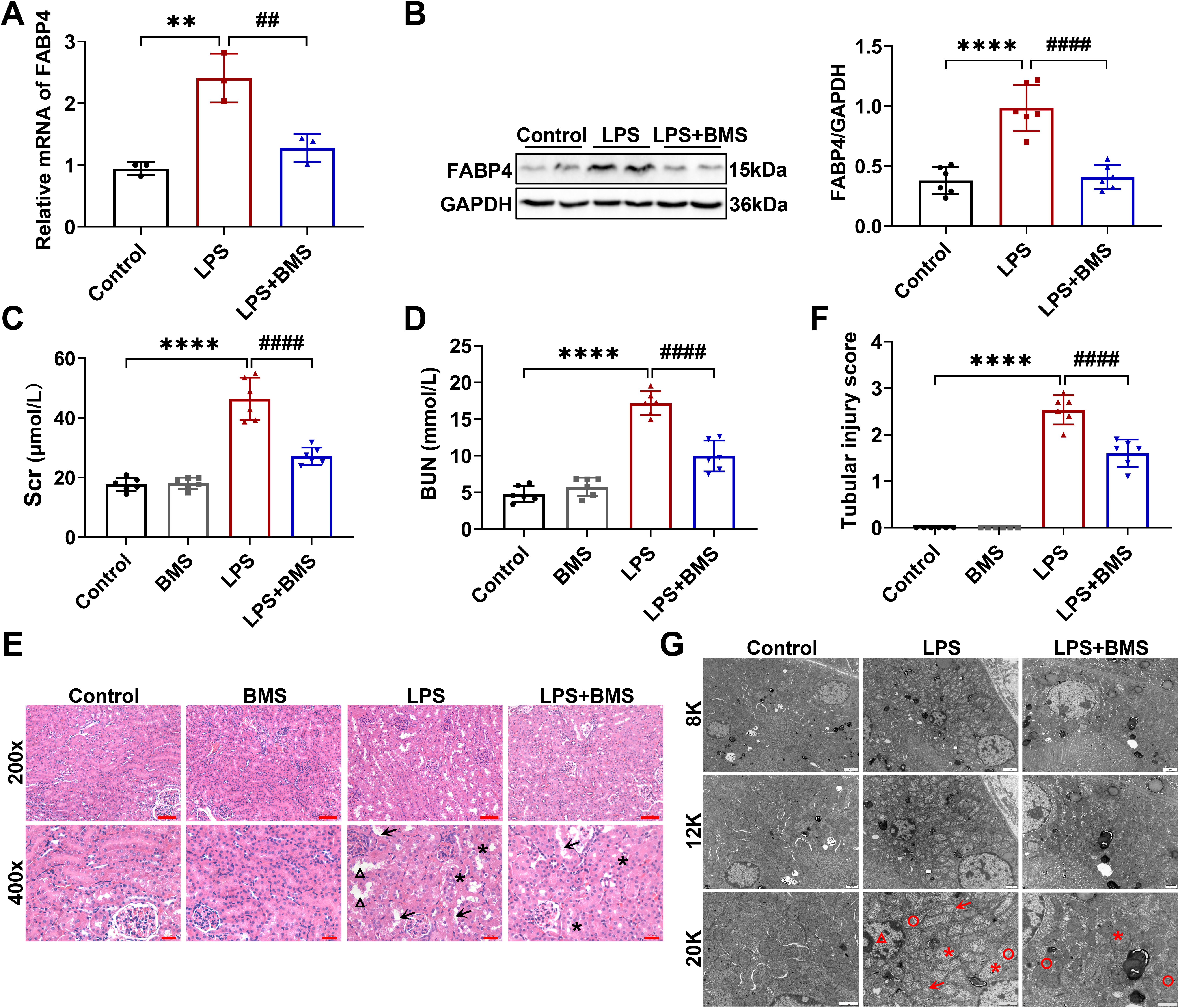


**Fig. S2 FABP4 inhibitor BMS309403 treatment ameliorated LPS-induced septic AKI in mice.** C57BL/6J and BMS309403 (BMS)-treated C57BL/6J mice (male, 8-10 weeks old) were injected intraperitoneally with 10 mg/kg LPS or 0.9 % saline and killed 16 h later. (**A**) Renal FABP4 mRNA expression in mice measured by quantitative real-time PCR (n = 3). (**B**) Renal FABP4 protein expression determined by western blotting, quantified by densitometry and normalized with GAPDH (n = 6). (**C**) Serum creatinine (Scr) and (**D**) BUN levels in different groups of mice (n = 6). (**E**) Representative micrographs of HE staining (200×, scale bar = 50 μm; 400×, scale bar = 20 μm) and (**F**) tubular injury scores of kidney tissues (n = 6). Triangle: tubular dilatation; Asterisk: tubular swelling; Arrow: loss of brush border. (**G)** Representative subcellular structures of renal tubular epithelial cells (RTECs) from different groups of mice collected by transmission electron microscope (TEM) (8000×, scale bar = 2 μm; 12000×, scale bar = 1 μm; 20000×, scale bar = 1 μm). Triangle: chromosome condensation; Asterisk: mitochondrial swelling; Circle: mitochondrial cristate fused; Arrow: mitochondrial cristate disappeared. All data are represented as mean ± SD; ^**^*P* < 0.01, ^****^*P* < 0.0001, versus Control; ^##^*P* < 0.01, ^####^*P* < 0.0001, versus LPS.

**Fig. S3 TLR4 deficiency or TLR4 inhibitor TAK242 treatment attenuated CLP-induced septic AKI in mice.** C57BL/6J, TAK242-treated C57BL/6J, TLR4 KO, and WT mice (male, 8-10 weeks old) were subjected to CLP or sham surgery and killed 16 h later. (**A**) Serum creatinine (Scr) and (**B**) BUN concentrations in different groups of mice (n = 6). (**C**) mRNA expression of NGAL and KIM1 measured by quantitative real-time PCR in renal tissues (n = 3). (**D**) Representative HE staining images (200×, scale bar = 50 μm; 400×, scale bar = 20 μm) and (**E**) tubular injury scores of kidney tissues (n = 6). Triangle: tubular dilatation; Asterisk: tubular swelling; Circle: cast formation; Arrow: loss of brush border. (**F**) Representative electron micrographs of renal tubular epithelial cells (RTECs) from different groups of mice (8000×, scale bar = 2 μm; 12000×, scale bar = 1 μm; 20000×, scale bar = 1 μm). Triangle: chromosome condensation; Asterisk: mitochondrial swelling; Circle: mitochondrial cristate fused; Arrow: mitochondrial cristate disappeared. All data are displayed as mean ± SD; ^****^*P* < 0.0001 for WT CLP versus WT Sham, or for CLP versus Sham; ^###^*P* < 0.001, ^####^*P* < 0.0001 for TLR4-KO CLP versus WT CLP, or for CLP+TAK242 versus CLP.


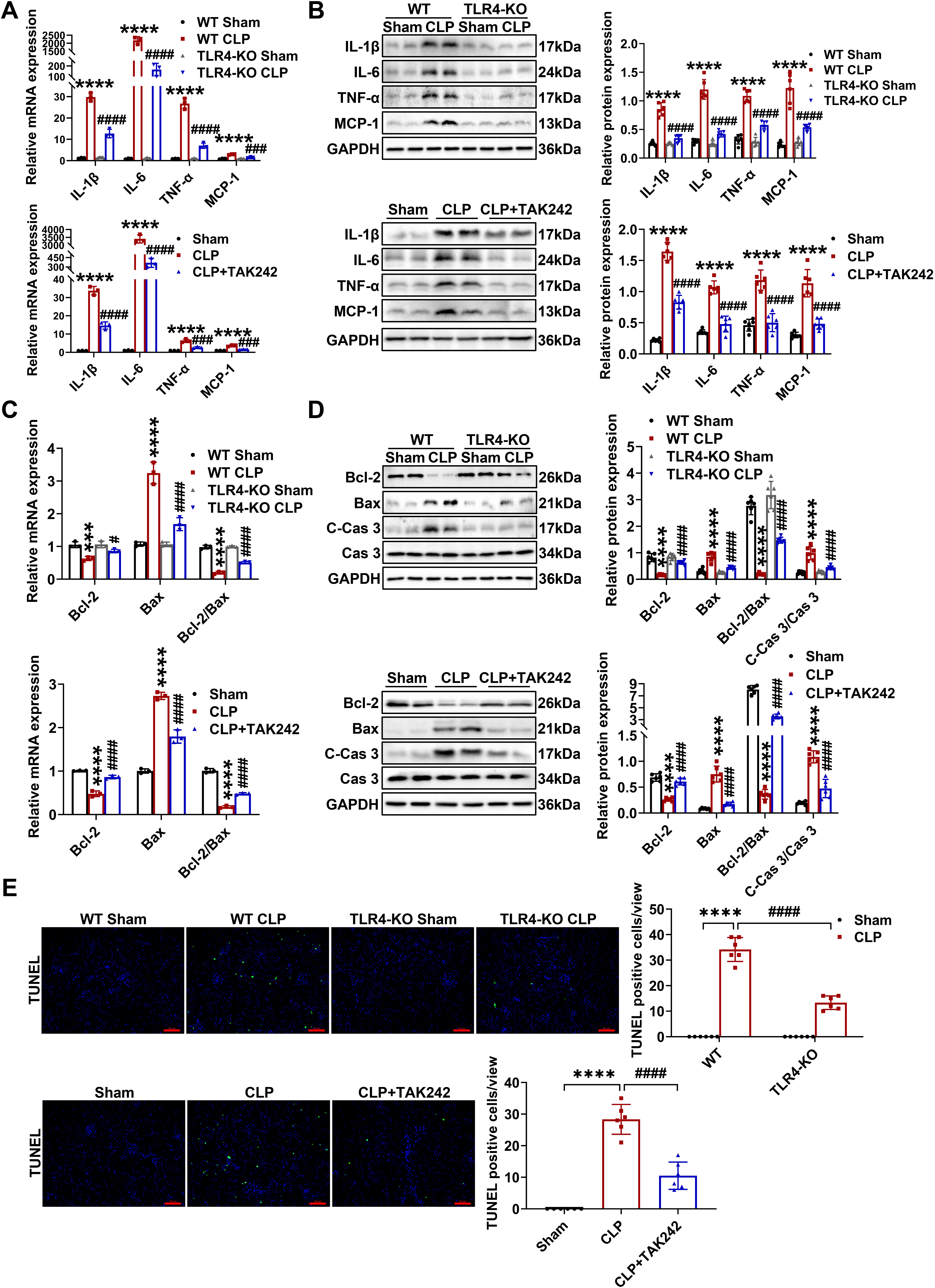


**Fig. S4 TLR4 deficiency or TLR4 inhibitor TAK242 treatment alleviated CLP-induced kidney inflammation and apoptosis in mice.** C57BL/6J, TAK242-treated C57BL/6J, TLR4 KO, and WT mice (male, 8-10 weeks old) were subjected to CLP or sham surgery and killed 16 h later. (**A**) The mRNA levels of inflammatory cytokines including IL-1β, IL-6, TNF-α, and MCP-1 in kidney tissues examined by quantitative real-time PCR (n = 3). (**B**) The protein levels of inflammatory cytokines including IL-1β, IL-6, TNF-α, and MCP-1 in the kidneys determined by western blotting and quantified by densitometry (n = 6). (**C**) The mRNA levels of apoptotic markers including Bcl-2 and Bax in renal tissues measured by quantitative real-time PCR (n = 3). (**D**) The protein levels of apoptotic markers including Bcl-2, Bax, Caspase 3 (Cas 3), and Cleaved-Caspase 3（C-Cas 3）in the kidneys analyzed by western blotting and quantified by densitometry (n = 6). (**E**) Representative micrographs of TUNEL staining (200×, scale bar = 50 μm) and number of TUNEL-positive cells in renal cortex (n = 6). Data are shown as mean ± SD; ^***^*P* < 0.001, ^****^*P* < 0.0001 for WT CLP versus WT Sham, or for CLP versus Sham; ^#^*P* < 0.05, ^###^*P* < 0.001, ^####^*P* < 0.0001 for TLR4-KO CLP versus WT CLP, or for CLP+TAK242 versus CLP.


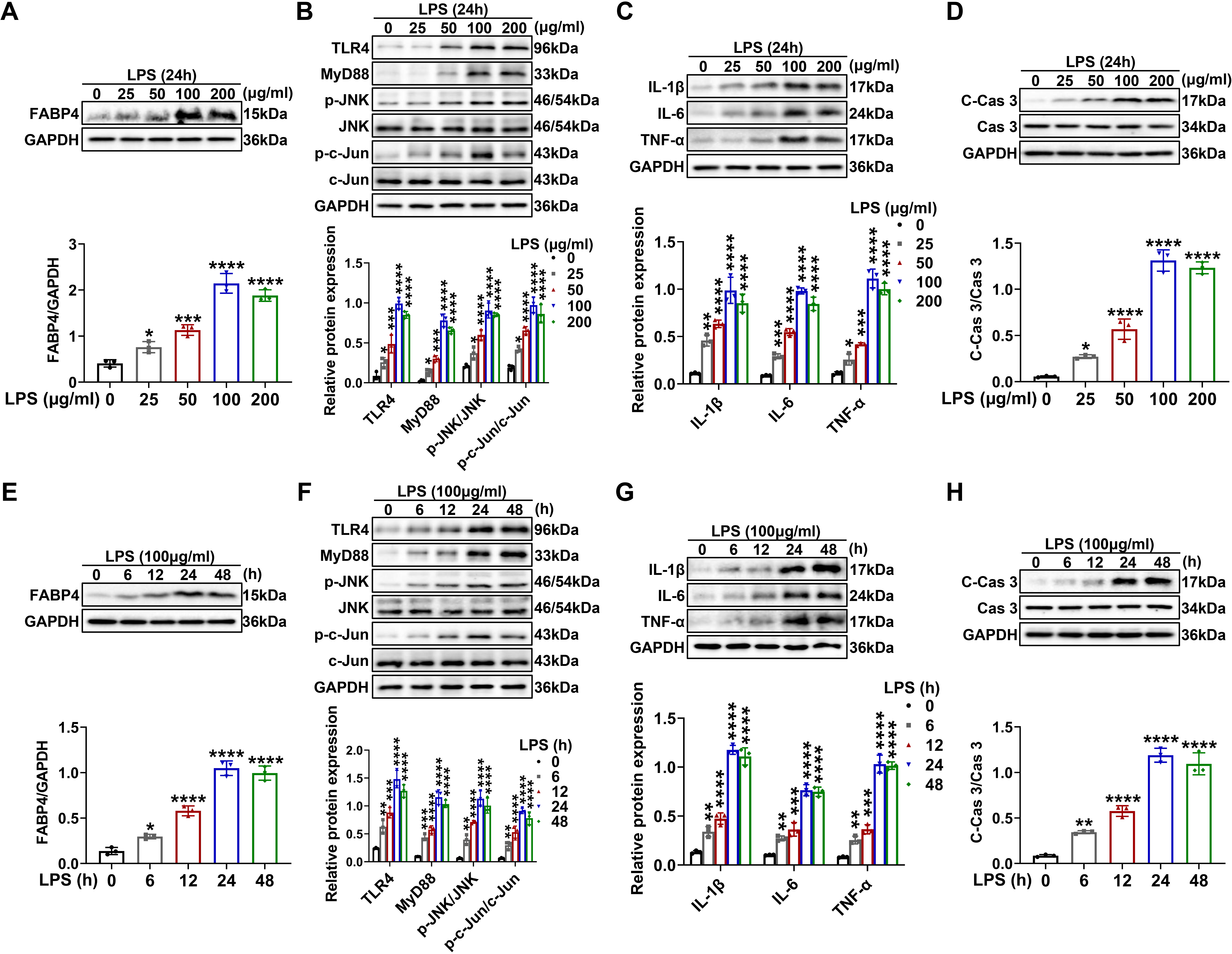


**Fig. S5 Effect of doses and timing of LPS stimulation in TCMK-1 cells.** TCMK-1 cells were treated with 0-200 μg/ml LPS for 0-48 h. Western blotting and densitometry quantification of **(A, E**) FABP4, and **(B, F**) TLR4, MyD88, p-JNK, JNK, p-c-Jun, c-Jun, and **(C, G**) inflammatory cytokines including IL-1β, IL-6, TNF-α, and **(D, H**) apoptotic markers including Caspase 3 (Cas 3) and Cleaved-Caspase 3（C-Cas 3) in TCMK-1 cells. All data are represented as mean ± SD (n = 3); ^*^*P* < 0.05, ^**^*P* < 0.01, ^***^*P* < 0.001, ^****^*P* < 0.0001 versus LPS (μg/ml) 0 group or LPS (h) 0 group.


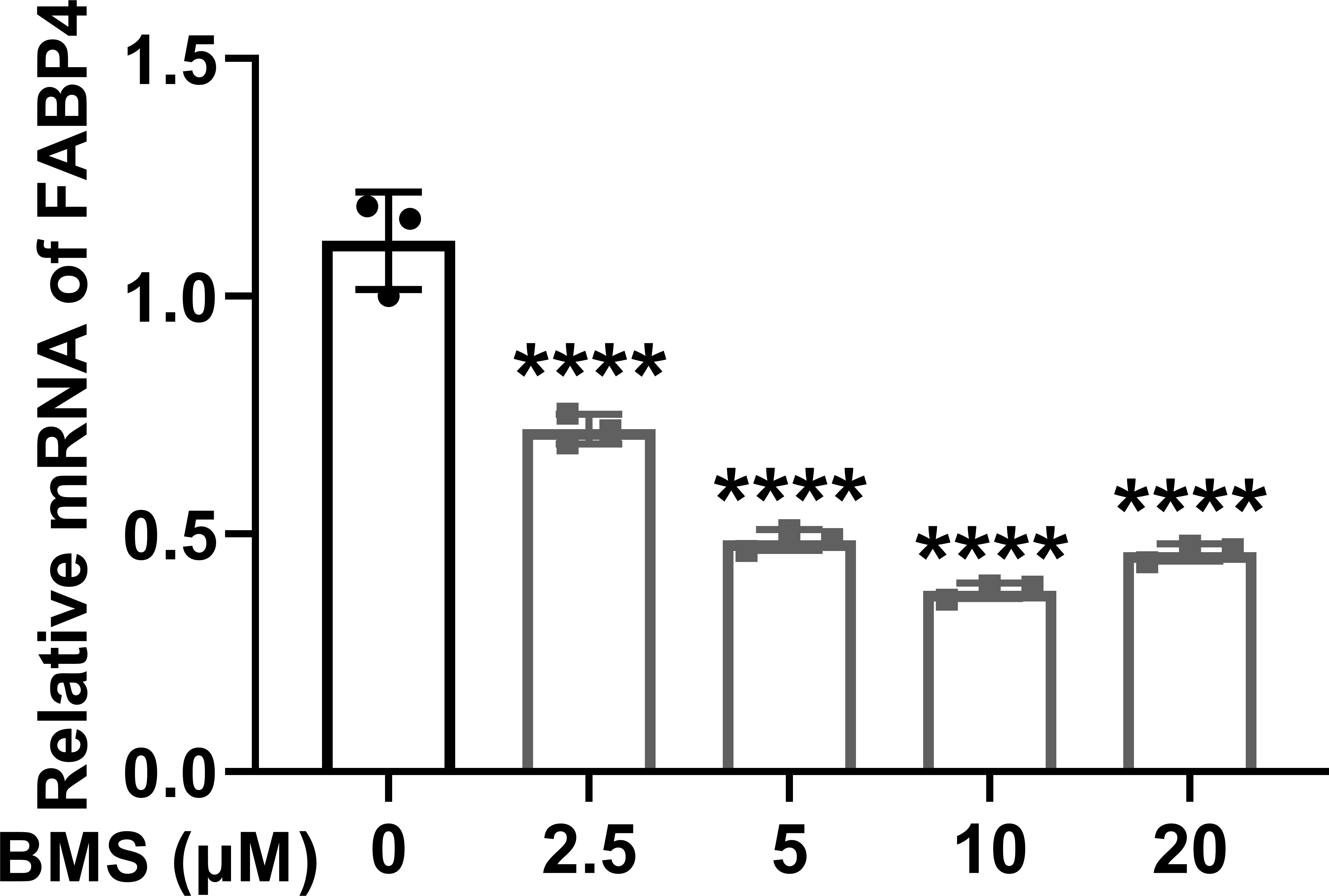


**Fig. S6 Effect of doses of FABP4 inhibitor BMS309403 treatment on FABP4 mRNA expression in TCMK-1 cells.** Quantitative real-time PCR analysis of FABP4 in TCMK-1 cells treated with 0-20 μM BMS309403 (BMS) for 24 h. Data are shown as mean ± SD (n = 3); ^****^*P* < 0.0001 versus BMS (μM) 0 group.


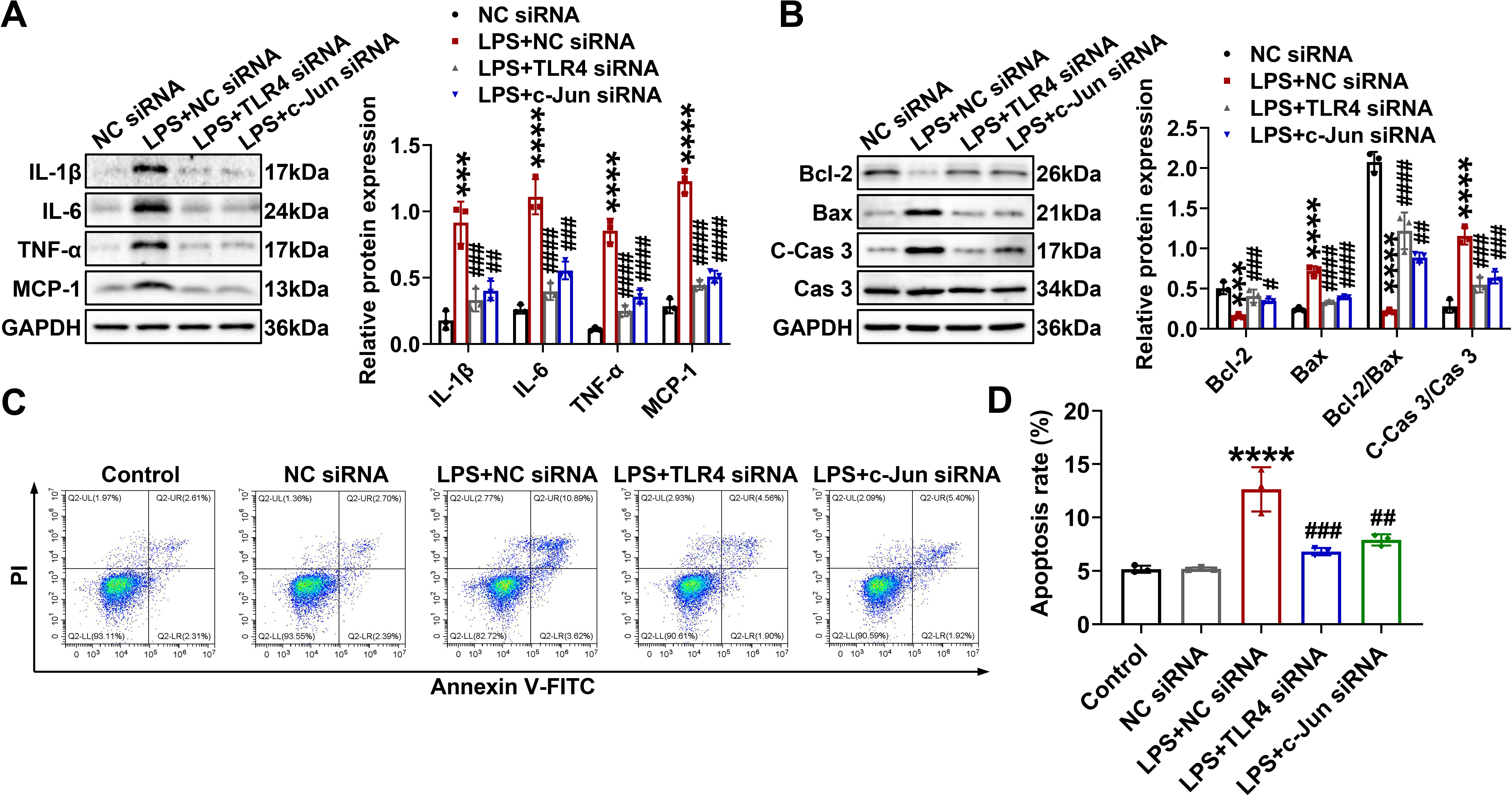


**Fig. S7 TLR4 or c-Jun knockdown inhibited inflammation and apoptosis in LPS-stimulated TCMK-1 cells.** TCMK-1 cells were transfected with negative control (NC) siRNA, TLR4 siRNA, or c-Jun siRNA for 24 h and then treated with 100 μg/ml LPS for another 24 h. Western blotting and densitometry quantification of (**A**) inflammatory cytokines including IL-1β, IL-6, TNF-α, and MCP-1, and (**B**) apoptotic markers including Bcl-2, Bax, Caspase 3 (Cas 3), and Cleaved-Caspase 3（C-Cas 3) in TCMK-1 cells. (**C**) Representative flow cytometric plots of TCMK-1 cell apoptosis and (**D**) quantification of apoptosis rate. All data are displayed as mean ± SD (n = 3); ^***^*P* < 0.001, ^****^*P* < 0.0001 versus NC siRNA; ^#^*P* < 0.05, ^##^*P* < 0.01, ^###^*P* < 0.001, ^####^*P* < 0.0001 versus LPS+NC siRNA.

# 3. Supplementary Tables

**Table S1.** Primary antibodies used in the experiments.

| **Name** | **Company** | **Catalog Number** |
| --- | --- | --- |
| Anti-FABP4 | Proteintech Group, Chicago, USA | 12802-1-AP |
| Anti-TLR4 | Abcam, MA, USA | Ab13556 |
| Anti-MyD88 | Affinity Biosciences, [Changzhou](D:/software/Dict/8.5.3.0/resultui/html/index.html#/javascript:;), China | AF5195 |
| Anti- phospho-JNK | HuaAn Biotechnology, [Hangzhou](D:/software/Dict/8.5.3.0/resultui/html/index.html#/javascript:;), China | ET1609-42 |
| Anti-JNK | HuaAn Biotechnology, [Hangzhou](D:/software/Dict/8.5.3.0/resultui/html/index.html#/javascript:;), China | RT1550 |
| Anti- phospho-c-Jun | HuaAn Biotechnology, [Hangzhou](D:/software/Dict/8.5.3.0/resultui/html/index.html#/javascript:;), China | ET1608-4 |
| Anti-c-Jun | HuaAn Biotechnology, [Hangzhou](D:/software/Dict/8.5.3.0/resultui/html/index.html#/javascript:;), China | ET1608-3 |
| Anti-IL-1β | Cell Signaling Technology, MA, USA | 12242S |
| Anti-IL-6 | HuaAn Biotechnology, [Hangzhou](D:/software/Dict/8.5.3.0/resultui/html/index.html#/javascript:;), China | EM170414 |
| Anti-TNF-α | Affinity Biosciences, [Changzhou](D:/software/Dict/8.5.3.0/resultui/html/index.html#/javascript:;), China | AF7014 |
| Anti-MCP-1 | Cell Signaling Technology, MA, USA | 2029S |
| Anti-Bcl-2 | Affinity Biosciences, [Changzhou](D:/software/Dict/8.5.3.0/resultui/html/index.html#/javascript:;), China | AF6139 |
| Anti-Bcl-XL | Affinity Biosciences, [Changzhou](D:/software/Dict/8.5.3.0/resultui/html/index.html#/javascript:;), China | AF6414 |
| Anti-BAX | Affinity Biosciences, [Changzhou](D:/software/Dict/8.5.3.0/resultui/html/index.html#/javascript:;), China | AF0120 |
| Anti-Cleaved caspase-3 | Cell Signaling Technology, MA, USA | 9579S |
| Anti-Caspase-3 | Proteintech Group, Chicago, USA | 66470-2-Ig |
| Anti-GAPDH | Zenbioscience, [Che](D:/software/Dict/8.5.3.0/resultui/html/index.html#/javascript:;)ngdu, China | 200306-7E4 |

| **Mouse Gene** | **Sequence** |
| --- | --- |
| F-FABP4 | CCGCAGACGACAGGA |
| R-FABP4 | CTCATGCCCTTTCATAAACT |
| F-TLR4 | GTCAGTGTGATTGTGGTATCC |
| R-TLR4 | ACCCAGTCCTCATTCTGACTC |
| F-MyD88 | TCATGTTCTCCATACCCTTGGT |
| R-MyD88 | AAACTGCGAGTGGGGTCAG |
| F-c-Jun | CCTTCTACGACGATGCCCTC |
| R-c-Jun | GGTTCAAGGTCATGCTCTGTTT |
| F-NGAL | GCAGGTGGTACGTTGTGGG |
| R-NGAL | CTCTTGTAGCTCATAGATGGTGC |
| F-KIM1 | ACATATCGTGGAATCACAACGAC |
| R-KIM1 | ACTGCTCTTCTGATAGGTGACA |
| F-IL-1β | TGGGCCTCAAAGGAAAGAAT |
| R-IL-1β | CAGGCTTGTGCTCTGCTTGT |
| F-IL-6 | ACAACCACGGCCTTCCCTACTT |
| R-IL-6 | CACGATTTCCCAGAGAACATGTG |
| F-TNF-α | ACCCTCACACTCAGATCATCTTC |
| R-TNF-α | TGGTGGTTTGCTACGACGT |
| F-MCP-1 | CATCCACGTGTTGGCTCA |
| R-MCP-1 | GATCATCTTGCTGGTGAATGAGT |
| F-Bcl-2 | TGTGAGGACCCAATCTGGAAA |
| R-Bcl-2 | TTGCAATGAATCGGGAGTTG |
| F-BAX | GATCAGCTCGGGCACTTTAG |
| R-BAX | TTGCTGATGGCAACTTCAAC |
| F-GAPDH | GTATGACTCCACTCACGGCAAA |
| R-GAPDH | GGTCTCGCTCCTGGAAGATG |

**Table S2.** Primer sequences used in real-time PCR analysis.
